# Modular Composition of Gene Transcription Networks

**DOI:** 10.1371/journal.pcbi.1003486

**Published:** 2014-03-13

**Authors:** Andras Gyorgy, Domitilla Del Vecchio

**Affiliations:** 1 Department of Electrical Engineering and Computer Science, Massachusetts Institute of Technology, Cambridge, Massachusetts, United States of America; 2 Department of Mechanical Engineering, Massachusetts Institute of Technology, Cambridge, Massachusetts, United States of America; Princeton University, United States of America

## Abstract

Predicting the dynamic behavior of a large network from that of the composing modules is a central problem in systems and synthetic biology. Yet, this predictive ability is still largely missing because modules display context-dependent behavior. One cause of context-dependence is retroactivity, a phenomenon similar to loading that influences in non-trivial ways the dynamic performance of a module upon connection to other modules. Here, we establish an analysis framework for gene transcription networks that explicitly accounts for retroactivity. Specifically, a module's key properties are encoded by three retroactivity matrices: internal, scaling, and mixing retroactivity. All of them have a physical interpretation and can be computed from macroscopic parameters (dissociation constants and promoter concentrations) and from the modules' topology. The internal retroactivity quantifies the effect of intramodular connections on an isolated module's dynamics. The scaling and mixing retroactivity establish how intermodular connections change the dynamics of connected modules. Based on these matrices and on the dynamics of modules in isolation, we can accurately predict how loading will affect the behavior of an arbitrary interconnection of modules. We illustrate implications of internal, scaling, and mixing retroactivity on the performance of recurrent network motifs, including negative autoregulation, combinatorial regulation, two-gene clocks, the toggle switch, and the single-input motif. We further provide a quantitative metric that determines how robust the dynamic behavior of a module is to interconnection with other modules. This metric can be employed both to evaluate the extent of modularity of natural networks and to establish concrete design guidelines to minimize retroactivity between modules in synthetic systems.

## Author Summary

Biological modules are inherently context-dependent as the input/output behavior of a module often changes upon connection with other modules. One source of context-dependence is retroactivity, a loading phenomenon by which a downstream system affects the behavior of an upstream system upon interconnection. This fact renders it difficult to predict how modules will behave once connected to each other. In this paper, we propose a general modeling framework for gene transcription networks to accurately predict how retroactivity affects the dynamic behavior of interconnected modules, based on salient physical properties of the same modules in isolation. We illustrate how our framework predicts surprising and counter-intuitive dynamic properties of naturally occurring network structures, which cannot be captured by existing models of the same dimension. We describe implications of our findings on the bottom-up approach to designing synthetic circuits, and on the top-down approach to identifying functional modules in natural networks, revealing trade-offs between robustness to interconnection and dynamic performance. Our framework carries substantial conceptual analogies with electrical network theory based on equivalent representations. We believe that the framework we have proposed, also based on equivalent network representations, can be similarly useful for the analysis and design of biological networks.

## Introduction

The ability to accurately predict the behavior of a complex system from that of the composing modules has been instrumental to the development of engineering systems. It has been proposed that biological networks may have a modular organization similar to that of engineered systems and that core processes, or motifs, have been conserved through the course of evolution and across different contexts [1], [2], [3], [4], [5]. In addition to having profound consequences from an evolutionary perspective, this view implies that biology can be understood, just like engineering, in a modular fashion [6]. To predict the behavior of a network from that of its composing modules, it is certainly desirable that the salient properties of modules do not change upon connection with other modules. This modularity property is especially important in a bottom-up approach to engineer biological systems, in which small systems are combined to create larger ones [7], [8].

Unfortunately, despite the fact that biological networks are rich of frequently repeated motifs, suggesting a modular organization, a module's behavior is often affected by its context [9]. Context-dependence is due to a number of different factors. These include unknown regulatory interactions between the module and its surrounding systems; various effects that the module has on the cell network, such as metabolic burden [10], effects on cell growth [11], and competition for shared resources [12]; and loading effects associated with known regulatory linkages between the module and the surrounding systems, a phenomenon known as retroactivity [13], [14]. As a result, our current ability of predicting the emergent behavior of a network from that of the composing modules remains limited. This inability is a central problem in systems biology and especially daunting for synthetic biology, in which circuits need to be re-designed through a lengthy and *ad hoc* process every time they are inserted in a different context [15].

In the phenomenon known as retroactivity, a downstream module perturbs the dynamic state of its upstream module in the process of receiving information from the latter [13], [14]. These effects are due to the fact that, upon interconnection, a species of the upstream module becomes temporarily unavailable for the reactions that make up the upstream module, changing the upstream module's dynamics. The resulting perturbations can have dramatic effects on the upstream module's behavior. For example, in experiments in gene circuits in *Escherichia coli*, a few fold ratio in gene copy number between the upstream module and the downstream target results in more than 40% change in the upstream module's response time [16]. More intriguing effects take place when the upstream module is a complex dynamical system such as an oscillator. In particular, experiments in transcriptional circuits *in vitro* showed that the frequency and amplitude of a clock's oscillations can be largely affected by a load [17] and computational studies on the genetic activator-repressor clock of [18] further revealed that just a few additional targets for the activator impose enough load to quench oscillations. Surprisingly, adding a few targets for the repressor can restore the stable limit cycle [19]. Retroactivity has also been experimentally demonstrated in signaling networks *in vitro* [20] and in the MAPK cascade *in vivo* [21]. In particular, it was shown in [19] that a few fold ratio between the amounts of the upstream and downstream system's proteins can lead to more than triple the response time of the upstream system.

In this paper, we provide a quantitative framework to accurately predict how and the extent to which retroactivity will change a module's temporal dynamics for general gene transcription networks and illustrate the implications on a number of recurrent network motifs. We demonstrate that the dynamic effects of loading due to interconnections can be fully captured by three retroactivity matrices. The first is the internal retroactivity, which accounts for loading due to intramodular connections. We illustrate that due to internal retroactivity, negative autoregulation can surprisingly slow down the temporal response of a gene as opposed to speeding it up, as previously reported [22]; perturbations applied at one node can lead to a response at another node even in the absence of a regulatory path from the first node to the second, having consequences relevant for network identification techniques (e.g., reviewed in [23]); and an oscillator design can fail even in the presence of small retroactivity. The other two matrices, which we call scaling and mixing retroactivity, account for loading due to intermodular connections. We illustrate that because of the scaling retroactivity, the switching characteristics of a genetic toggle switch can be substantially affected when the toggle switch is inserted in a multi-module system such as that proposed for artificial tissue homeostasis in [24]. The interplay between scaling and internal retroactivity plays a role in performance/robustness trade-offs, which we illustrate considering the single-input motif [5]. Using these retroactivities, we further provide a metric establishing the robustness of a module's behavior to interconnection. This metric can be explicitly calculated as a function of measurable biochemical parameters, and it can be used both for evaluating the extent of modularity of natural networks and for designing synthetic circuits modularly.

Our work is complementary to but different from studies focusing on partitioning large transcription networks into modules using graph-theoretic approaches [13], [25], [26]. Instead, our main objective is to develop a general framework to accurately predict both the quantitative and the qualitative behavior of interconnected modules from their behavior in isolation and from key physical properties (internal, scaling, and mixing retroactivity). In this sense, our approach is closer to that of disciplines in biochemical systems analysis, such as metabolic control analysis (MCA) [27], [28]. However, while MCA is primarily focused on steady state and near-equilibrium behavior, our approach considers global nonlinear dynamics evolving possibly far from equilibrium situations.

This paper is organized as follows. We first introduce a general mechanistic model for gene transcription networks to explain the physical origin of retroactivity and to formulate the main question of the paper (System Model and Problem Formulation). We then provide the two main results of the paper (Results). These are obtained by reducing the mechanistic model through the use of time scale separation (leading to models of the same dimension as those based on Hill functions), in which only macroscopic parameters and protein concentrations appear. In these reduced models, the retroactivity matrices naturally arise, whose practical implications are illustrated on five different application examples.

### System Model and Problem Formulation

We begin by introducing a standard mechanistic model for gene transcription networks, which includes protein production, decay, and reversible binding reactions between transcription factors (TFs) and promoter sites, required for transcriptional regulation. Specifically, transcription networks are usually viewed as the input/output interconnection of fundamental building blocks called transcriptional components. A transcriptional component takes a number of TFs as inputs, and produces a single TF as an output. The input TFs form complexes with promoter sites in the transcriptional component through reversible binding reactions to regulate the production of the output TF, through the process of gene expression (for details, see Methods). To simplify the notation, we treat gene expression as a one-step process, neglecting mRNA dynamics. This assumption is based on the fact that mRNA dynamics occur on a time scale much faster than protein production/decay [1]. In addition to this, including mRNA dynamics is not relevant for the study of retroactivity, and would yield only minor changes in our results (see Methods).

Within a transcription network, we identify a transcriptional component with a *node*. Consequently, a transcription network is a set of interconnected nodes in which node 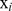 represents the transcriptional component producing TF 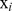. There is a directed edge from node 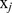 to 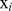 if 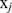 is a TF regulating the activity of the promoter controlling the expression of 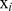 [29], in which case we call 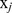 a *parent* of 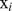. Activation and repression are denoted by 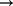 and 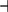, respectively. *Modules* are a set of connected nodes. Modules communicate with each other by having TFs produced in one module regulate the expression of TFs produced in a different module. When a node 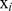 is inside the module, we call the corresponding TF 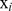 an *internal* TF, while when node 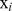 is outside the module we call the corresponding TF 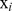 an *external* TF. Further, we identify external TFs that are parents to internal TFs as *inputs* to the module. Let 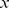, 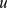 and 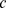 denote the concentration vector of internal TFs, inputs and TF-promoter complexes, respectively. According to [30], we can write the dynamics of the module as
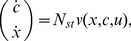
(1)where 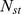 is the stoichiometry matrix and 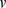 is the reaction flux vector. The reactions are either protein production/decay or binding/unbinding reactions. Therefore, we partition 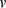 into 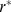 and 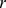, representing the reaction flux vectors corresponding to production/decay and binding/unbinding reactions, respectively (see Methods). We assume that the DNA copy number is conserved, therefore, we can rewrite (1) as
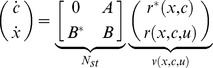
where the upper left block matrix in 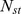 is the zero matrix as DNA is not produced/degraded. As a result, with 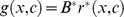 we obtain
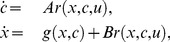
(2)which we call the *isolated dynamics* of a module.

Next, consider the case when the module is inserted into a network, which we call the *context* of the module. We represent all the quantities related to the context with an overbar. Let 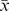 and 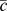 denote the concentration vector of TFs and promoter complexes of the context, respectively. Furthermore, denote by 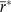 and 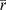 the reaction flux vectors corresponding to production/decay and binding/unbinding reactions between TFs and promoters in the context of the module, respectively. Then, the dynamics of the species in the module (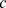 and 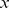) and in the context (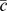 and 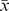) can be written as
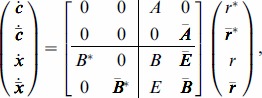
(3)where the upper left block matrix is zero as DNA is assumed to be a conserved species. Furthermore, since 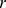 and 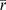 encapsulate the binding/unbinding reactions in the module and in its context, respectively, the off-diagonal block matrices in the upper right block matrix are zero. Similarly, as 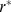 and 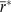 encapsulate the production/decay reactions in the module and its context, respectively, the off-diagonal block matrices in the lower left block matrix are zero. Finally, the stoichiometry matrix 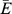 represents how internal TFs of the module participate in binding/unbinding reactions in the context of the module (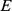 can be interpreted similarly).

**Figure 1:**
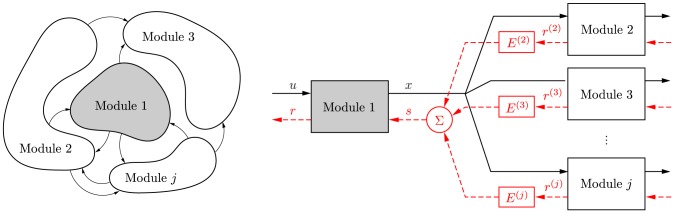
The dynamics of a module depend on the module's context. Downstream modules change the dynamics of an upstream module by applying a load. The effect of this load is captured by the retroactivity to the output 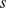 of the upstream module, which is the weighted sum of the retroactivity to the input 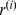 of the downstream modules.

With 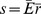 describing the effective rate of change of 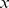 due to intermodular binding reactions, we obtain
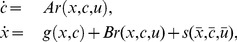
(4)which we call the *connected dynamics* of a module. We refer to 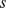 as the *retroactivity to the output* of the module, encompassing retroactivity applied to the module due to the context of the module. Similarly, we call 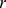 the *retroactivity to the input* of a module, representing retroactivity originating inside the module. The general interconnection of a group of modules can be treated similarly (Figure 1).

**Figure 2:**
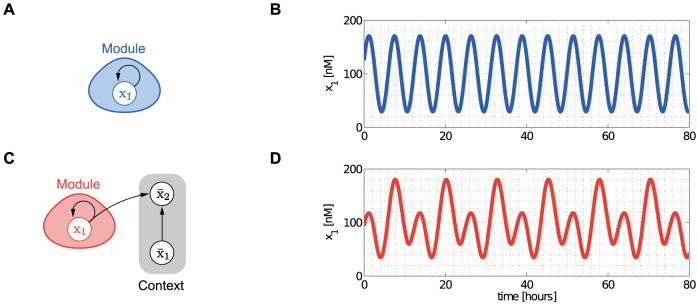
The context (downstream system) affects the behavior of the module (upstream system). (A) The module in isolation. (B) The module in isolation displays sustained oscillations. (C) The module connected to its context. (D) Upon interconnection with its context, the dynamics of the module change due to the retroactivity 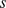 from its context, since some of the molecules of 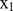 are involved in binding reactions at node 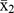. As a result, those molecules are not available for reactions in the module, and the output of the module is severely changed. For details on the system and parameters, see Supporting Text S1.

As an example of the implications of retroactivity 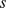 on the module's dynamic behavior, consider Figure 2. For the purpose of illustration, assume that 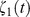 and 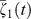, external inputs to 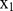 and 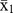 (see Methods), are periodic (in general, they can be arbitrary time-varying signals). When the module is not connected to its context (Figure 2A), its output is periodic (Figure 2B). Upon interconnection with its context (Figure 2C), due to the retroactivity to the output 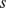 applied by the context, the output of the module changes significantly (Figure 2D). Hence, connection with the context leads to a dramatic departure of the dynamics of the module from its behavior in isolation. This example illustrates that retroactivity 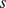 significantly alters the dynamic behavior of modules after interconnection, therefore, it cannot be neglected if accurate prediction of temporal dynamics is required. Unfortunately, model (4) provides little analytical insight into how measurable parameters and interconnection topology affect retroactivity.

The aim of this paper is to provide a model that captures the effects of retroactivity, unlike standard regulatory network models of the same dimension based on Hill functions [1]. Specifically, we seek a model that explicitly describes the change in the dynamics of a module once it is arbitrarily connected to other modules in the network. This model is only a function of measurable biochemical parameters, TF concentrations, and interconnection topology.

## Results

We first characterize the effect of intramodular connections on an isolated module's dynamics. We then analytically quantify the effects of intermodular connections on a module's behavior. Finally, we determine a metric of robustness to interconnection quantifying the extent by which the dynamics of a module are affected by its context. We demonstrate the use of our framework and its implications on network motifs taken from the literature.

The main technical assumptions in what follows are that (a) there is a separation of time scale between production/degradation of proteins and the reversible binding reactions between TFs and DNA, and that (b) the corresponding quasi-steady state is locally exponentially stable. Assumption (a) is justified by the fact that gene expression is on the time scale of minutes to hours while binding reactions are on the second to subsecond time scale [3]. Assumption (b) is implicitly made any time Hill function-based models are used in gene regulatory networks. In addition to these technical assumptions, to simplify notation, we model gene expression as a one-step process, however, a more detailed description of transcription/translation would not yield any changes to the main results (see Methods).

### Effect of Intramodular Connections

Here, we focus on a single module without inputs and describe how retroactivity among nodes, modeled by 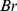 in (2), affects the module's dynamics. To this end, we provide a model that well approximates the isolated module dynamics, in which only measurable macroscopic parameters appear, such as dissociation constants and TF concentrations. We then present implications of this model for negative autoregulation, combinatorial regulation and the activator-repressor clock of [18].

**Figure 3:**
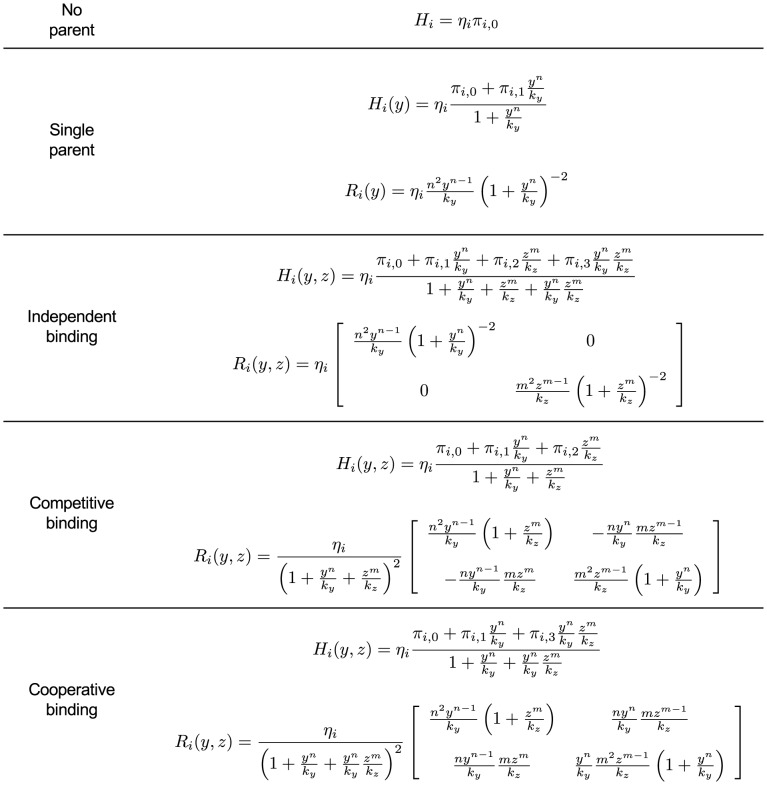
Hill function and retroactivity of node 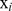 for the most common binding types. If node 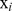 has no parents, its node retroactivity is not defined. In the single parent case, node 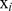 has one parent, 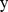 binding as an 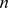-multimer with dissociation constant 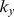. In the case of independent, competitive and cooperative binding, node 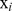 has two parents, 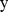 and 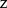, binding as multimers with multimerization factors 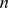 and 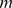, respectively, together with dissociation constants 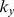 and 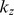, respectively. The total concentration of the promoter of 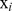 is denoted by 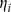. The production rates 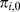, 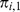, 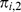 and 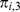 correspond to the promoter complexes without parents, with 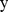 only, with 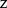 only, and with both 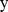 and 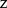, respectively. For details, see Supporting Text S3.

Employing assumptions (a)–(b), we obtain the first main result of the paper as follows. Let 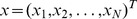 denote the vector of concentrations of internal TFs, then the dynamics
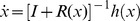
(5)well approximate the dynamics of 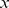 in (2) in the isolated module with
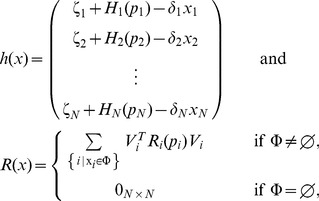
(6)where 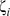 represents external perturbations to 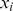 (inducer, noise, or disturbance, 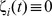 unless specified otherwise), 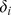 is the decay rate of 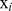, and 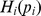 is the Hill function modeling the production rate of 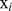, regulated by the parents 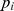 of 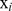. We call 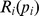 the *retroactivity* of node 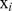. For the most common binding types, Figure 3 shows the expressions of 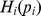 and 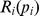 (for their definition, see Methods). The binary matrix 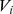 has as many columns as the number of nodes in the module, and as many rows as the number of parents of 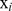, such that its 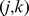 element is 1 if the 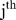 parent of 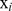 is 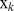, otherwise the entry is zero. That is, an entry in the following matrix
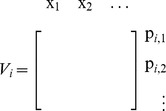
is 1 if the species indexing the corresponding row and column are the same, otherwise the entry is zero, yielding 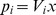. Finally, 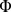 is the set of nodes having parents from inside the module. For the derivation of this result, see Theorem 1 in Supporting Text S2.

We call 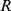 the *internal retroactivity* of the module as it describes how retroactivity among the nodes internal to the module affects the isolated module dynamics. When 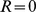, we have 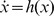, the commonly used Hill function-based model for gene transcription networks [3]. It is possible to show that 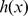 represents the rate of change of total (free and bound) TFs (see Supporting Text S2). Hence, (6) describes how changes in the total concentration of TFs 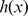 relate to changes 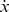 in the concentration of free TFs. Specifically, to change the concentration of free TFs by one unit, the module has to change the total concentration of TFs by 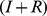 units, as 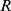 units are “spent on” changing the concentration of bound TFs. Having 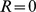 implies that the module's effort on affecting the total concentration of TFs is entirely spent on changing the concentration of free TFs. By contrast, 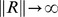 implies that no matter how much the total concentration of TFs changes, it is not possible to achieve any changes in the free concentration of some of the TFs. Therefore, the internal retroactivity 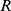 describes how “stiff” the module is against changes in 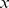 due to loading applied by internal connections.

The entries of 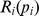 have the following physical interpretation. Consider first a module with the autoregulated node 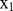, that is, 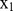 has a single parent: itself. The retroactivity of node 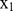 is 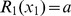, where 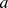 is given in Figure 3. In this case, we obtain 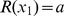 by (6), so that (5) yields 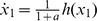. Hence, the greater 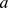, the harder to change the concentration of free 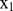 by changing its total concentration (the “stiffer” the node), and the temporal dynamics of 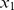 become slower. The retroactivity 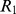 of node 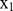 can be increased by increasing its DNA copy number 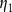 or by decreasing the dissociation constant 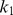 of 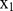. For a node with two parents, we provide the explicit formula for 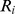 in Figure 3 in the case of the most frequent binding types, so that here we simply write
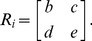
(7)The diagonal entries 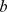 and 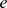 in (7) can be interpreted similarly to 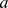, while the off-diagonal entries can be interpreted as follows. Having 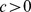 means that the second parent facilitates the binding of the first, whereas 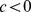 represents blockage (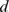 can be interpreted similarly with the parents having reverse roles). Therefore, we have 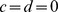 in the case of independent binding (Figure 3), as the parents bind to different sites. By contrast, we have 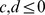 in the case of competitive binding (Figure 3), since the parents are competing for the same binding sites, forcing each other to unbind. Following a similar reasoning, we obtain 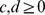 in the case of cooperative binding (Figure 3). Notice that 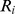 is scaled by the total concentration of promoter 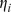, which can be changed, for example, in synthetic circuits by changing the plasmid copy number.

### Practical Implications of Intramodular Connections

In order to illustrate the effects of intramodular connections, we consider three recurrent network motifs in gene transcription networks: (i) negative autoregulation of a gene, (ii) combinatorial regulation of a gene by two TFs, and (iii) the activator-repressor clock of [18].

#### Negative autoregulation

One of the most frequent network motifs in gene transcription networks is negative autoregulation, as over 40% of known *Escherichia coli* TFs are autorepressed [29]. Earlier studies concluded that negative autoregulation makes the response of a gene faster [22]. Here, we demonstrate that in the case of significant retroactivity, negative autoregulation can actually slow down the response of a gene. To this end, consider a module consisting of the single node 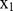, and analyze first the case when its production is constitutive with promoter concentration 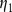, production rate constant 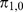 and decay rate 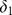. Then, the dynamics of 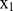 are given by 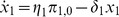.

In the case of negative autoregulation, 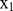 has itself as the only parent. Let 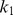 denote the dissociation constant of 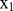 and assume it binds as a monomer repressing its own production (so that 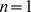 and 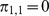 in Figure 3). According to Figure 3, we have 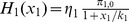 and 
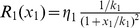
 together with 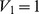 and 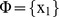, yielding from (6) 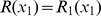 and 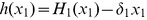, so that (5) results in
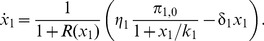
(8)This expression indicates that negative autoregulation yields two changes in the dynamics. First, protein production changes from 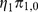 to the Hill function 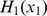. Second, the dynamics are premultiplied by 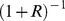, which is the effect of internal retroactivity.

**Figure 4:**
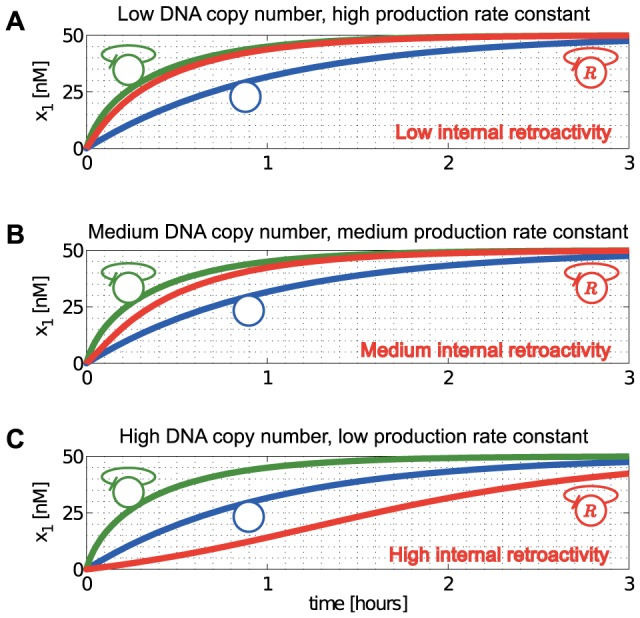
Negative autoregulation can make the temporal response slower. Time response at a steady state fixed at 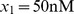. The red and blue plots denote the cases with and without negative autoregulation, respectively, whereas the green plot represents the case of negative autoregulation neglecting retroactivity (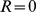 in (8)). Simulation parameters are 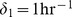, 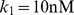, together with 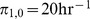, 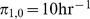, 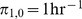 for A, B and C, respectively. To carry out a meaningful comparison between the unregulated and regulated systems, we compare the response time of systems with the same steady state. To do so, we pick the same value of 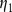 in the case of the regulated systems (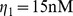, 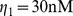, 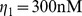 for A, B and C, respectively), but a different one for for the unregulated system (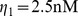, 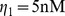, 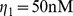 for A, B and C, respectively), such that the steady states match (see Methods for parameter ranges). Decreasing 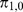 (lower production rate constant) while increasing 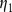 (higher DNA copy number) results in slower response, as internal retroactivity increases.

As it was shown in [22], the response time of the regulated system without retroactivity is smaller than that of the unregulated system. When considering internal retroactivity, however, the response time increases, as the absolute value of 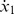 decreases with increased 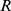 according to (8). Specifically, the response time with 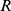 is greater than without 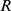 since 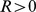. That is, while the Hill function makes the response faster, internal retroactivity has an antagonistic effect, so that negative autoregulation can render the response slower than that of the unregulated system, as illustrated in Figure 4. Furthermore, if 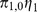 is kept constant, the response time of both the unregulated (blue) and the regulated system without retroactivity (green) remain the same, together with the steady states. By contrast, increasing 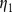 (and decreasing 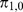) makes the internal retroactivity 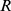 greater (since 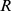 is proportional to 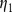), while the contribution of the Hill function remains unchanged. As a result, the response of the regulated system with retroactivity (red) becomes slower as we increase 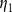 (and decrease 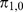). This is illustrated in Figure 4 with different 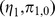 pairs. Note that 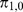 can be decreased, for example, by decreasing the ribosome binding site (RBS) strength, whereas 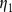 can be increased by increasing the gene copy number.

#### Combinatorial regulation

**Figure 5:**
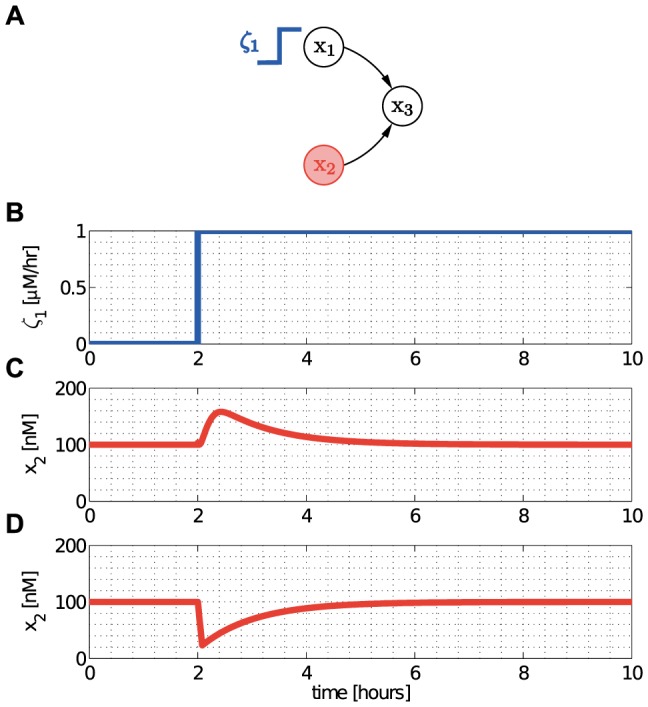
Nodes can become coupled via common downstream targets. (A) Node 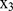 has two parents: 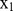 and 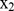, without a regulatory path between them. (B) Perturbation 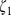 applied to 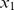. (C) In the case of competitive binding, increasing the concentration of free 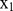 yields more of 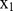 bound to the promoter of 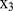, forcing some of the molecules of 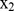 to unbind, thus increasing the free concentration 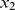. Consequently, 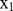 acts as if it were an activator of 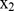. (D) By contrast, in the case of cooperative binding, when the binding of 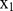 must precede that of 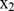, pulses in 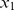 yield pulses of the opposite sign in 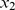. Consequently, 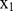 acts as if it were a repressor of 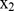. Simulation parameters are: 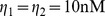, 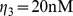, 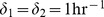, 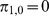, 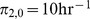, 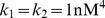, and both 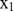 and 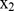 bind as tetramers.

As a second example, we consider a single gene co-regulated by two TFs (Figure 5A). This topology appears in recurrent network motifs, such as the feedforward-loop, the bi-fan and the dense overlapping regulon [5]. Here, we show that a perturbation introduced in one of the parents (blue in Figure 5A) can affect the concentration of the other parent (red node in Figure 5A), even in the absence of a regulatory path between the two.

Referring to (5)–(6), note that 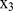 is the only node with parents (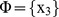), so that 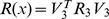. Using (6) with
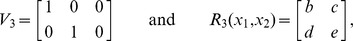
where the entries of 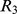 are given in Figure 3 (depending on the binding type at 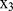) together with 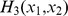, the dynamics in (5) take the form
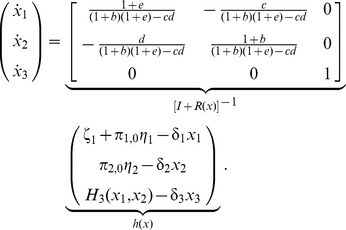
This expression implies that unless 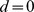, a perturbation 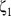 (Figure 5B) in 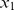 yields a subsequent perturbation in 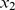. In the case of independent binding, we have 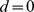, and as a result, no perturbation is observed in 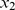. In the case of competitive binding, instead, we have 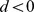, so that perturbations 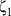 in 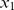 yield perturbations of the same sign in 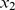, that is, 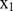 acts as if it were an activator of 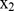 (Figure 5C). In the case of cooperative binding, instead, we have 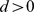. As a result, perturbations in 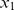 yield perturbations in 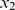 of opposite sign (Figure 5D), which implies that 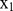 behaves as if it were a repressor of 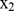. As 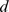 is proportional to 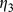 (Figure 3), higher DNA copy number for 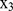 yields greater pulses in 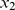 subsequent to an equal perturbation in 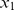. Interestingly, if we view 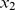 as the output of the module, the module has the adaptation property with respect to its input 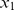 (or 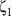). That is, retroactivity enables to respond to sudden changes in input stimuli, while adapting to constant stimulus values.

#### Activator-repressor clock

**Figure 6:**
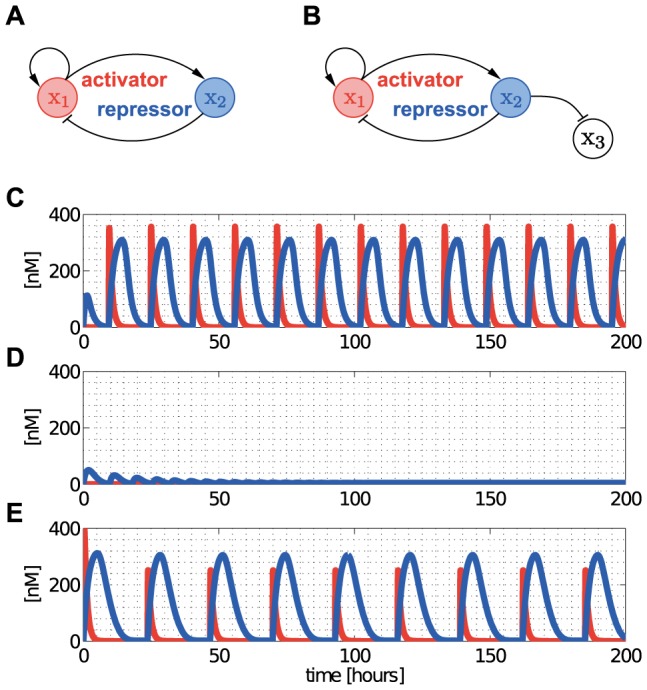
Neglecting internal retroactivity could falsely predict that the activator-repressor clock will display sustained oscillations. (A) The module consists of the activator protein 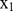 (dimer) and the repressor protein 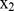 (tetramer), with dissociation constants 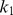 and 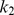, respectively. (B) An extra node 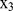 is introduced as a target for the repressor. (C) Without accounting for internal retroactivity, the module in A exhibits sustained oscillations. (D) When internal retroactivity is included for the module in A, however, the equilibrium point is stabilized and the limit cycle disappears. (E) Oscillations can be restored by applying a load on the repressor (module in B) with concentration 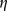, so that the repressor dynamics are slowed down. Simulation parameters: 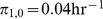, 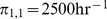, 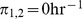, 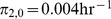, 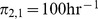, 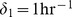, 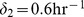, 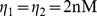, 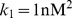, 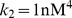 and 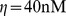.

One common clock design is based on two TFs, one of which is an activator and the other is a repressor [18], [31], [32]. Here, we illustrate the effect of internal retroactivity on the functioning of the clock design of [18] depicted in Figure 6A. In particular, 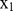 activates the production of both TFs, whereas 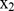 represses the production of 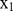 through competitive binding. Consequently, the network topology is captured by the binary matrices 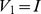 and 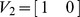, whereas 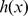 and 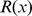 can be constructed by considering 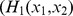, 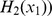 and 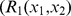, 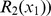, respectively, in Figure 3. Here, we write 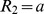, while the entries of 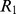 are denoted by 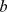, 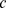, 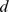 and 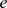, as in (7). Then, we obtain that (5) takes the form
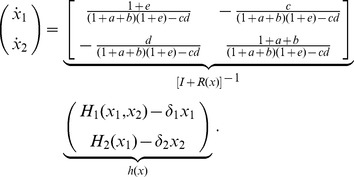
(9)It was previously shown [33] that the principle for the clock to oscillate is that the activator dynamics are sufficiently faster than the repressor dynamics (so that the unique equilibrium point is unstable). Equation (9) shows that the activator and repressor dynamics are slowed down asymmetrically (diagonal terms in 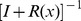), and that they become coupled (off-diagonal terms in 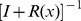, 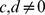), because of internal retroactivity. In particular, in the case when 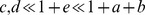, the activator would slow down compared to the repressor. Based on the principle of functioning of the clock, we should expect that this could stabilize the equilibrium point, quenching the oscillations as a consequence. In fact, oscillations disappear even if the circuit is assembled on DNA with a single copy (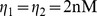), as it can be observed in Figure 6D. Therefore, accounting for internal retroactivity is particularly important in synthetic biology during the design process when circuit parameters and parts are chosen for obtaining the desired behavior. An effective way to restore the limit cycle in the clock, yielding sustained oscillations, is to render the repressor dynamics slower with respect to the activator dynamics. This can be obtained by adding extra DNA binding sites for the repressor [19], as shown in Figure 6B. In fact, in this case, we have 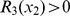 given in Figure 3, which, due to (5), will yield the following change in (9): instead of 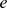, we will have 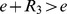, rendering the dynamics of the repressor slower with respect to the activator dynamics. As a result, the equilibrium point becomes unstable, restoring the limit cycle, verified by simulation in Figure 6E. Further studies on specific systems have investigated the effects of TF/promoter binding on the dynamics of loop oscillators, such as the repressilator [34].

### Effect of Intermodular Connections

After investigating how retroactivity due to intramodular connections affect a single module's dynamics, we next determine how the dynamics of a module change when the module is inserted into its context. To this end, we first extend the model in (5) to the case in which the module has external TFs as inputs. Hence, let 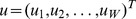 denote the concentration vector of TFs external to the module. With this, we obtain that the dynamics
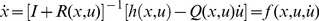
(10)well approximate the dynamics of 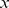 in (2) with
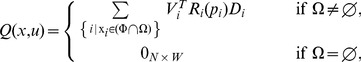
(11)where 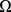 is the set of nodes having parents from outside the module (external TFs), and the binary matrix 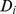 has as many columns as the number of inputs of the module, and as many rows as the number of parents of 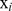, such that its 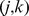 element is 1 if the 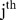 parent of 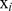 is 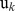, otherwise the entry is zero. That is, an entry in the following matrix
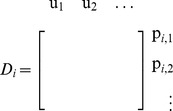
is 1 if the species indexing the corresponding row and column are the same, otherwise the entry is zero, yielding 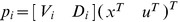. Furthermore, note that in the presence of input 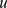, both 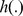 and 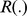 given in (6) depend on 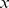 and 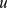, as some of the parents of internal TFs are external TFs. For the derivation of this result, see Theorem 2 in Supporting Text S2.

Before stating the main result of this section, we first provide the interpretation of 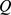. Recall that 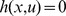 implies that the total concentrations of internal TFs are constant. In this case, (10) reduces to 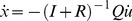, where 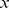 is the concentration vector of free internal TFs. This means that the concentrations of free internal TFs can still be changed subsequent to changes in the external TFs (input), despite the fact that the total concentration (free and bound) of internal TFs remains unaffected. Therefore, 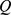 captures the phenomenon by which external TFs force internal TFs to bind/unbind, for instance, by competing for the same binding sites. Having 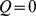 means that external TFs do not affect the binding/unbinding of internal TFs, which is the case, for example, when all bindings are independent. Thus, we call 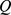 the *external retroactivity* of the module.

The main result of this section describes how the context of a module affects the module's dynamics due to retroactivity. Specifically, we consider the module of interest and we represent the rest of the network, the module's context, as a different module. As previously, we use the overbar to denote that a quantity belongs to the context. With this, we obtain that the dynamics
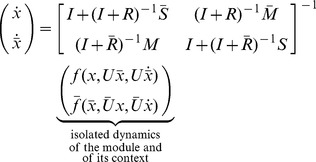
(12)well approximate the dynamics of 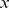 and 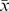 in (4) in the module connected to the context with
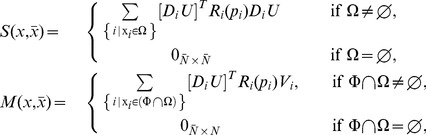
(13)where 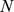 and 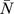 denote the number of nodes in the module and in the context, respectively. Furthermore, the binary matrix 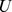 has as many rows as the number of inputs of the module, and as many columns as the number of nodes in the context, such that its 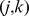 element is 1 if the 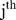 input of the module is the 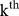 internal TF of the context (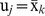), otherwise the entry is zero. That is, an entry in the following matrix
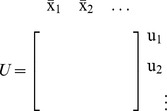
is 1 if the species indexing the corresponding row and column are the same, otherwise the entry is zero, yielding 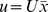. The quantities corresponding to the context, that is, 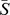, 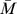 and 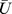 are defined similarly with the only difference that variables with and without overbar have to be swapped (for instance, 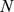 and 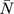 have to be swapped in (13)). For the derivation of this result, see Theorem 3 in Supporting Text S2.

We next provide the interpretation of the scaling and mixing retroactivity. The reduced order model (12) describes how retroactivity between the module and the context affects each other's dynamics. Note that zero matrices 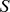, 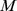, 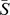 and 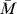 lead to no alteration in the dynamics upon interconnection. To further deepen the implications of these matrices and their physical meaning, note that when 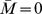, the dynamics of the module after interconnection become
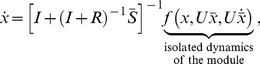
(14)that is, 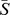 determines how the isolated dynamics of the module get “scaled” upon interconnection. Therefore, we call 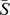 the *scaling retroactivity* of the context, accounting for the loading that the context applies on the module as some of the TFs of the module are taken up by promoter complexes in its context (we obtain 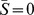 if nodes in the context do not have parents in the module, that is, if 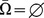). Since the dynamics of the context enter into the module's dynamics through 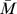, we call 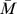 the *mixing retroactivity* of the context, referring to the “mixing” of the dynamics of the module and that of its context. The mixing retroactivity 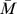 establishes how internal TFs force external TFs to bind/unbind, so that 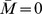 can be ensured if the binding of parents from the module is independent from that of the parents from the context. This holds if nodes in the context are not allowed to have parents in both the module and in the context (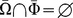). When 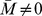, a perturbation applied in the context can result in a response in the upstream module, even without TFs in the context regulating TFs in the module, leading to a counter-intuitive transmission of signals from downstream (context) to upstream (module).

With this, we can explain the simulation results in Figure 2D by analyzing 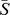 and 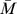 for the system in Figure 2C. Let 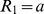 and let 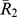 be defined as in (7), where 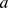, 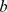, 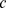, 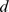 and 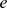 are given in Figure 3. Then, we have 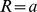 by (13) and 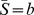 and 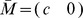 by (6). Hence, expression (12) yields
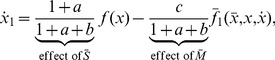
(15)describing the dynamics of 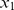 upon interconnection with its context, where 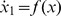 and 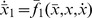 describe the dynamics of 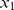 and 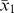, respectively, when the module and the context are not connected to each other. If 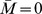, then (15) reduces to
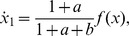
that is, the context rescales the dynamics of the module. The smaller 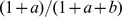, that is, the greater the scaling retroactivity 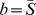, the greater the effect of this scaling. Note that since the scaling factor is smaller than 1 (unless the scaling retroactivity is zero, i.e., 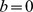), the effect of the scaling retroactivity of the context in this case is to make the temporal dynamics of the module slower.

Once 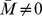, in addition to this sclaing effect, the dynamics of the context appear in the dynamics of the module (Figure 2D). Referring to (15), we can quantify the effect of the context on the module, considering the ratio 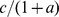. The greater 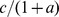, that is, the greater 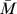, the stronger the contribution of the context compared to that of the module to the dynamics of the module upon interconnection. Here, both 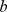 and 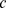 increase, for instance, with the copy number of 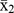 (Figure 3).

Connecting the module to its context such that 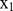 and 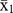 are competing for the same binding sites is less desirable than employing independent binding, as the dynamics of the context (downstream system) can suppress the dynamics of the module (upstream system). Dismantling the dynamics of the module will “misinform” other downstream systems in the network that are regulated by 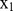. From a design perspective, multi-module systems should be designed and analyzed such that the modules have zero mixing retroactivity. This can be achieved, for instance, by avoiding non-independent binding at the interface nodes (at 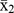 in Figure 2B). However, since completely independent binding can be hard to realize in the case of combinatorial regulation, nodes integrating signals from different modules should not be placed into the input layer (nodes having parents from other modules), yielding 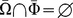. This can be achieved by introducing an extra input node in the downstream module (see Supporting Figure S1).

Next, we quantify the difference between the isolated and connected module behavior. In particular, we provide a metric of the change in the dynamics of a module upon interconnection with its context, dependent on 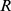 and 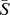 and under the assumption that 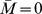 (obtained, for instance, by avoiding mixing parents from the module and the context). The isolated dynamics of the module can be well approximated by the reduced order model 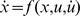 in (10), and let 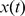 denote its solution. Once we connect the module to its context, its dynamics change according to (14), which we write as 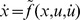 and let 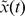 denote the corresponding solution. Using the sub-multiplicative property of the induced 2-norm, we have that the percentage change of the dynamics upon interconnection can be bounded from above as follows:
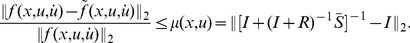
(16)Furthermore, with 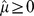 independent of 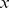 and 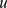, such that 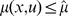, we obtain that
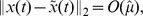
that is, 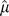 provides an upper bound on the percentage change in the dynamics of the module, and on the difference in the trajectories of the module upon interconnection with its context. Furthermore, by using the properties of the induced 2-norm, we obtain that we can pick
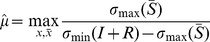
provided that 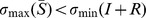 for all 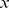 and 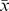, where 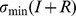 and 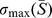 denote the smallest singular value of 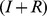 and largest singular value of 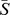, respectively. For the mathematical derivations, see Supporting Text S2. This suggests that the module becomes more robust to interconnection by increasing 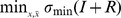 or by decreasing 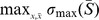.

Such a metric can be used both in the analysis and in the design of complex gene transcription networks as follows. Given any network and a desired module size 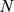 (number of nodes within the module), we can identify the module that has the least value of 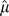, that is, the module with the greatest guaranteed robustness to interconnection. Furthermore, we can also evaluate existing partitionings based on other measures (e.g., edge betweenness [35], its extension to directed graphs with nonuniform weights [36], round trip distance [25] or retroactivity [37]) with respect to robustness to interconnection. From a design point of view, one can design multi-module systems such that internal, scaling and mixing retroactivities allow for low values of 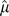, leading to modules that behave almost the same when connected or isolated.

### Practical Implications of Intermodular Connections

We next illustrate the effect of intermodular connections on the dynamics of interconnected modules, considering both a synthetic genetic module that is being employed in a number of applications and a natural recurring network motif.

#### Toggle switch

**Figure 7:**
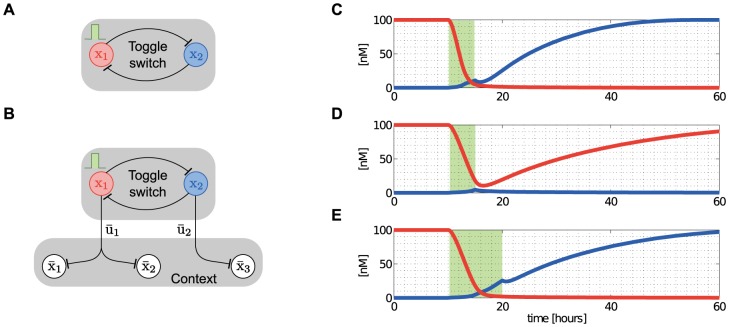
Effects of the context on the switching characteristics of the toggle switch. (A) The toggle switch in isolation. (B) The toggle switch connected to its context [40]. (C) A narrow pulse in 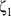 (input perturbation in 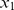, depicted in green) causes the isolated toggle to switch between the two stable equilibria. (D) When connected to the context, the same pulse is insufficient to yield a switch. (E) With a wider pulse, the switching is restored (however, dynamics are slower compared to the isolated case). Simulation parameters: both 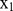 and 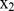 bind as dimers, 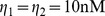, 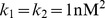, 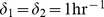, 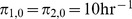, 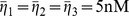, and the height of the input perturbation pulse is 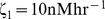.

Here, we consider the toggle switch of [38], a bistable system that can be permanently switched between two steady states upon presentation of a transient input perturbation. This module has been proposed for synthetic biology applications in biosensing (see, for example, [34], [39]). In this paper, we consider the toggle switch inserted into the context of the synthetic circuit for controlling tissue homeostasis as proposed in [24], and investigate how the context of the toggle affects its switching characteristics. Figure 7A illustrates the toggle switch in isolation, whereas Figure 7B shows the configuration when connected to the context [24]. Note that all nodes, both in the toggle switch and in its context, have a single parent. Therefore, 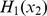, 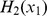, 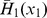, 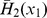, 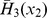, and similarly, 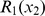, 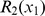, 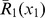, 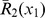, 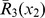 are given in Figure 3.

We first consider the model of the toggle switch when not connected to its context (Figure 7A). Since the toggle switch has no input, its isolated dynamics are described by (5), where 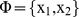, 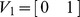 and 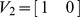 yield
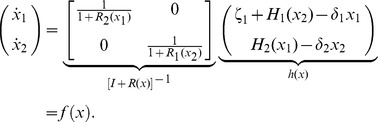
Next, we consider the toggle switch connected to its context (Figure 7B). As nodes in the toggle switch have no parents from outside it, we have 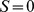 and 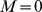 by (13). Nodes in the context have no parents in the context, leading to 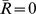 from (6), and to 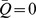, referring to (11). With this, the isolated dynamics of the context are given by
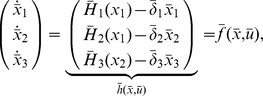
according to (10). The fact that nodes in the context do not have mixed parents from the toggle switch and from the context results in 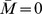 from (13). With 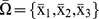, 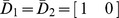, 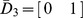, and 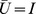 we obtain
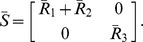
As a result, with 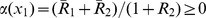 and 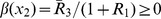, the dynamics of the toggle switch once connected to the context (Figure 7B) are given by
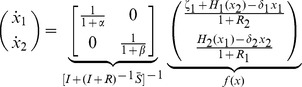
according to (12), so that the dynamics of 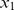 and 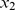 are unaffected if 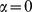 and 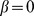, respectively. When 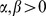, both the 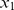 and 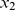 dynamics become slower upon interconnection, so that the response to an input stimulation will also be slower. As a consequence, upon removal of the stimulation, the displacement in the toggle state may not be sufficient to trigger a switch. This is illustrated in Figure 7C–D. In order to recover the switch, a wider pulse is required (Figure 7E) to compensate for the slow-down due to the context (also, note that the switching dynamics are slower than in the isolated case). As a result, even if the toggle had been characterized in isolation, it would fail to function as expected when inserted into its context. Note that we have 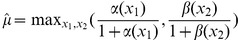, where 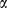 represents the amount of load on 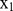 imposed by the context compared to that by the module, and 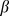 can be interpreted similarly. The greater 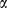 (or 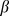), the slower the dynamics of 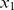 (of 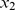) become upon interconnection with the context. Greater 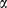 and 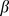 yield greater 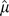, suggesting decreased robustness to interconnection, verified by the simulation results.

#### Single-input motif

**Figure 8:**
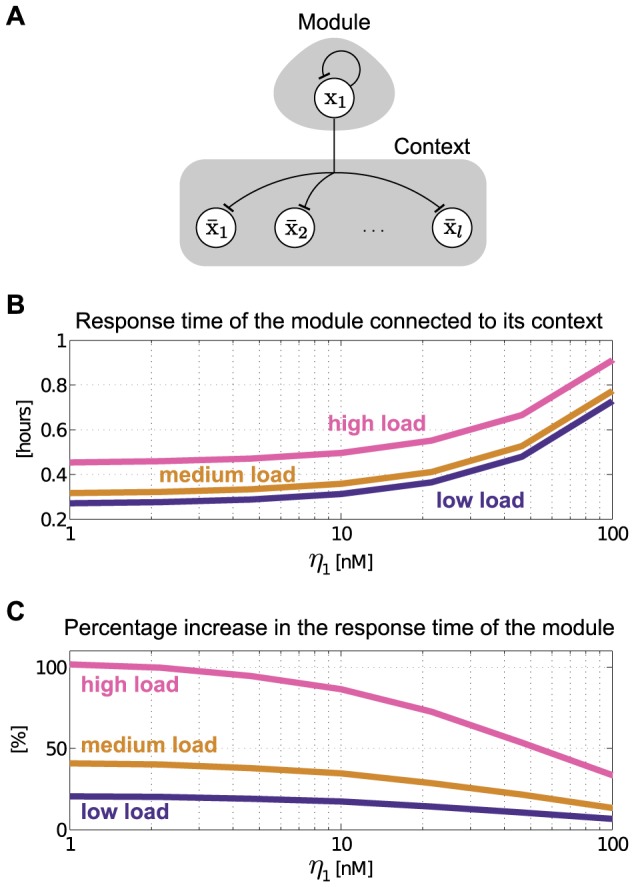
Internal retroactivity makes a module more robust to interconnection at the expense of speed. (A) The module consists of a single negatively autoregulated node, whereas the context comprises 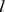 nodes repressed by the TF in the module. (B) The internal retroactivity 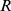 of the module increases with the DNA copy number 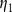 of 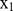. As a result, the module becomes slower as 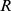 increases. (C) The percentage increase in the response time of the module decreases with 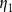, that is, internal retroactivity 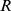 increases the robustness to interconnection. Simulation parameters: 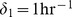, 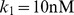 and 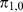 is changed such that 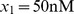 at the steady state. The context contains 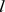 nodes each with DNA concentration 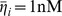 for 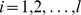 (low load: 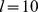; medium load: 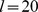; high load: 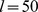). The response time is calculated as the time required to reach 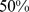 of the steady state value.

As a second example, we focus on a recurrent motif in gene transcription networks, called the single-input motif [5]. The single-input motif is defined by a set of operons (context) controlled by a single TF (module), which is usually autoregulated (Figure 8A). It is found in a number of instances, including the temporal program controlling protein assembly in the flagella biosynthesis [40]. Here, we show that the dynamic performance (speed) of the module and its robustness to interconnection with its context are not independent, and that this trade-off can be analyzed by focusing on the interplay between the internal retroactivity 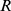 of the module and the scaling retroactivity 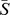 of the context.

The isolated dynamics of the module are given in (8), which we write here as 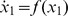. Furthermore, we have 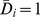 for 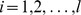 and 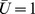, so that 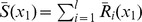 by (13), where 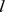 is the number of nodes in the context and 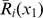 is given in Figure 3 (single parent). Consequently, upon interconnection, the dynamics of the module change according to (14) as
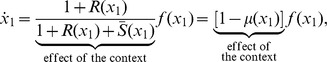
where 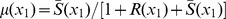 and equals the expression in (16). The smaller 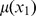, the more robust the module to interconnection. Note that 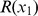 is proportional to 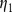, therefore, while increasing 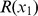 makes the module slower (Figure 8B), it also makes it more robust to interconnection (Figure 8C). It was previously shown that negative autoregulation increases robustness to perturbations [41]. Here, we have further shown that increasing the internal retroactivity 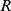 of the module provides an additional mechanism to increase robustness to interconnection, at the price of slower response. For a fixed steady state (the product of 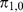 and 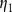 is held constant), smaller 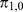 yields greater 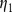, that is, increased 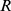 and, in turn, smaller 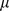. From a design perspective, if speed is a priority, one should choose a strong RBS with a low copy number plasmid, or alternatively, a promoter with high dissociation constant 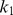. By contrast, if robustness to interconnection is central, a weak RBS with a high copy number plasmid (or with low 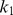) is a better choice. If both speed and robustness to interconnection are desired, other design approaches may be required, such as the incorporation of insulator devices, as proposed in other works [42].

#### Remark

**Figure 9:**
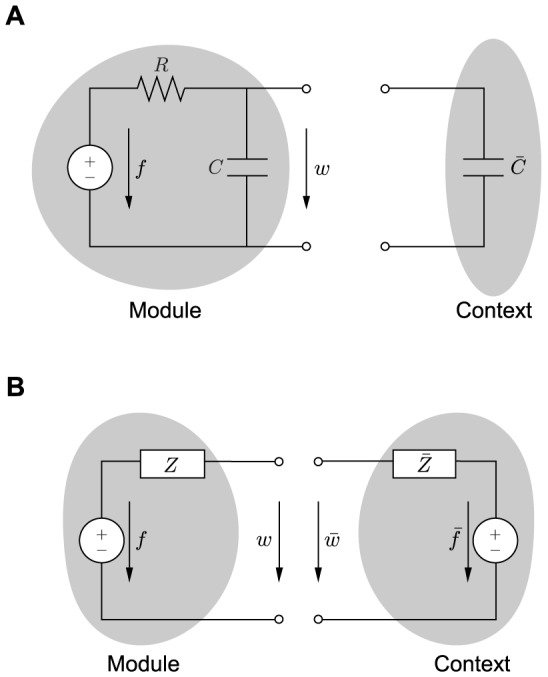
Analogy with electrical systems. (A) The module consists of the series interconnection of a voltage source 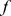, a resistor 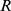 and a capacitor 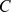. The speed of the module can be characterized by the time constant 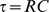, which increases upon interconnection with the context. The greater 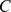, the slower the module in isolation, but the smaller the percentage change in its speed upon interconnection. (B) According to the fundamental theorem by Thevenin [48], any linear electrical network can be equivalently represented by a series interconnection of a voltage source and an impedance. As a result, a generic module consists of the series interconnection of a voltage source 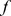 and an impedance 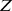, and similarly, any context can be represented with the series interconnection of 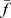 and 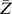.

The above presented trade-off between robustness to interconnection and dynamic performance can be observed also in electrical systems. To illustrate this, consider the electrical circuit in Figure 9A consisting of the series interconnection of a voltage source 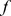, a resistor 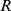 and a capacitor 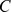, in which the output voltage is 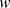. The speed of the circuit can be characterized by its time constant 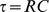: the greater 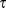, the slower the response. Upon interconnection with its context, represented by the capacitor 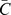, the time constant of the system changes to 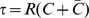, while the steady state remains the same. Note that the percentage change in 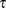 decreases with 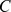, making the module more robust to interconnection, at the expense of slower response when isolated.

To further generalize the analogy between electrical systems and gene transcription networks [43], consider the electrical circuits in Figure 9B. When the module is not connected to its context, we have 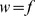 and 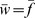, which changes to
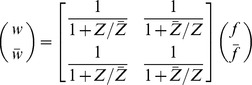
upon interconnection. This relationship is conceptually analogous to (12). That is, the module is robust to interconnection with its context if 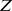 is small compared to 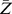, whereas the genetic module is robust to interconnection with its context if 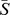 and 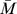 are “small” compared to 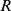. Therefore, 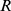 is conceptually analogous to 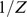 (output admittance), whereas 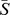 and 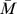 play a role similar to 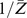 (input admittance).

## Discussion

In this paper, we have focused on retroactivity, one source of context-dependence, and demonstrated that the internal, scaling, and mixing retroactivity provide missing knowledge that captures loading effects due to intramodular and intermodular connections. The internal retroactivity quantifies the effect of intramodular load, applied by nodes within a module onto each other because of binding to promoter sites within the module. Given a module of interest, the effects of intramodular loading on the module's dynamics are captured by equations (5)–(6), in which one needs to replace the specific expressions of the Hill functions 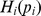 and node retroactivities 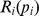 provided in Figure 3, and the binary matrices 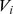 encoding the network topology. The scaling retroactivity accounts for the intermodular loading that the context applies on a module, due to having some TFs of the module bound to promoter sites belonging to the context. The mixing retroactivity couples the dynamics of the module and that of the context upon interconnection, and it is non-zero when TFs from different modules bind non-independently at promoter sites. The effects of intermodular loading are captured by equations (10)–(13). To obtain this description, it is sufficient to consider the Hill functions 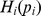 and node retroactivities 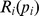 provided in Figure 3, together with the binary matrices 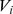, 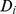 and 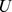 representing the network topology. In general, the effects of the retroactivity matrices tend to increase with increased DNA copy number and/or decreased dissociation constants.

We have illustrated that accounting for retroactivity reveals surprising dynamical properties of modules and, at the same time, can aid design. For example, negative autoregulation, depending on the gene copy number and production rates, can slow down the response of a system instead of speeding it up. A gene can respond to a perturbation applied to a different gene even in the absence of a regulatory path between the two genes. We have shown that this can occur when a group of TFs co-regulate common targets and these common targets are found in abundance. This type of motif, referred to as the dense overlapping regulon, is highly frequent in natural regulatory networks [1]. As a result, system identification techniques based on perturbation analysis [23] could erroneously identify non-existent regulatory linkages if retroactivity is not accounted for in the corresponding models. An activator-repressor clock on low copy DNA plasmid displays sustained oscillations when internal retroactivity is neglected, while oscillations are quenched once internal retroactivity is accounted for. However, by carefully adjusting the module's internal retroactivity through the addition of DNA load for the repressor, we can restore oscillations. A genetic toggle switch that can be flipped by a transient external stimulation requires a substantially longer stimulation to be flipped once it is connected to just few downstream targets. These facts are relevant, in particular, when designing synthetic biology circuits and multi-module systems.

Similar to synthetic systems, natural systems are subject to retroactivity. For example, clocks responsible for circadian rhythms have a large number of downstream targets [44], [45], which, in turn, may apply substantial load. This load can affect the amplitude and frequency of oscillations of the clock as well as the stability of the corresponding limit cycle. This suggests that natural systems may have evolved to use retroactivity in advantageous ways such as using it to properly tune the dynamic behavior of a module without changing the module's components. This hypothesis is further supported by the fact that there are a large number of TF binding sites on the chromosome that do not have a regulatory function [46], [47]. These sites have an impact on the temporal response of TFs, and could therefore be exploited by nature to further control the dynamics of gene regulation. More generally, retroactivity provides means for information to travel from downstream targets to upstream regulators, therefore establishing indirect connections. In highly interconnected topologies, this information transfer can result in previously unknown ways of realizing sophisticated functions. One such example that we have provided is the adaptation function that topologies such as the dense overlapping regulon can realize by virtue of having nodes co-regulate multiple downstream targets.

Based on the three retroactivity matrices, we provided a metric of robustness to interconnection, quantifying the percent change between the dynamics of a module in isolation and once connected to other modules. This metric is an explicit function of measurable parameters and becomes smaller when a module's internal retroactivity is large compared to the scaling retroactivity of the modules it connects to. This interplay may help uncover trade-offs in natural systems, providing a new angle for understanding natural principles of network organization. From an engineering perspective, we have provided quantitative design tools that can be employed in synthetic biology to appropriately match the internal and scaling retroactivity of connected circuits to preserve the circuits' behavior upon interconnection with different contexts. Our metric of robustness to interconnection further allows to evaluate the extent of modularity of a dynamical module, possibly enabling the discovery of previously unknown core processes. Our metric could be employed by currently available methods for partitioning networks into modules. Specifically, to evaluate connectivity, these methods rely on several metrics, for instance, edge betweenness [35], its extension to directed graphs with nonuniform weights [36], round trip distance [25] or retroactivity [37]. The metric of robustness to interconnection that we have introduced can enhance these methods by providing a way to evaluate modules on the basis of their functional robustness to interconnection in addition to distinguishing them at the connectivity level.

The framework that we have proposed carries substantial conceptual analogies with the electrical circuit theory established by Thevenin [48], which has been used for more than a hundred years to analyze and to design electrical networks. Within this theory, each circuit has an equivalent input and output impedance (conceptually analogous to the scaling/mixing and internal retroactivity, respectively), and an equivalent energy source (playing a role similar to the isolated module dynamics). This theory has been instrumental for answering key questions in the analysis and design of electrical networks including, for example, how the output of a circuit changes after it is interconnected in a network; how to design circuits to maximize the power transfer upon connection (impedance matching); and how to design circuits whose input/output response is unaffected by loads. We believe that the framework proposed in this paper can be used in a similar way for the analysis and design of gene regulatory networks.

Although our framework can be applied to a general gene transcription network, there are a number of aspects that it does not currently capture. These include post-translational protein modifications, such as phosphorylation, which are present in many regulatory networks and may potentially affect retroactivity. Including these will require to extend our framework to mixed gene transcription and signaling network models, leading to systems with multiple time scales. Furthermore, the transcription and translation processes use shared resources such as RNA polymerase and ribosomes, which may create couplings among unconnected circuits [17]. The dynamics of shared resources has not been included in our modeling framework and will be the focus of our future work.

## Methods

### Detailed Description of the System Model

The production of TF 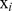 is regulated by its parents 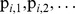: they bind to the promoter of 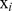, and form complexes 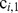, 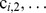 with the promoter. Each of these complexes, in turn, produce 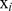 with a different rate, where we use a one-step production process encapsulating both transcription and translation [3]. As a result, the reactions we consider for node 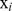 are
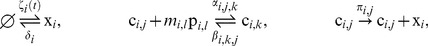
(17)modeling the following physical phenomena. We denote by 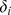 protein decay, whereas 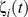 represents the production rate that may be due to external inputs or perturbations (inducer, noise or disturbance). The second reversible reaction in (17) describes the binding of parent 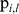 with multimerization factor 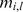 to promoter complex 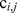 forming complex 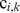, where 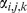 and 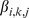 are the association and dissociation rate constants, respectively. Furthermore, each promoter complex 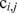 will contribute to the production of 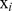 through the production rate constant 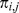, modeled by the third reaction in (17). This production rate constant is a lumped parameter that incorporates features such as the RBS strength and the promoter strength. Finally, we assume that the total concentration of the promoter, denoted by 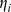, for each transcription component is conserved, so that 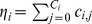, where 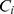 is the number of possible complexes formed with the promoter of 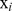. This concentration is proportional to the concentration of copies of the promoter, which can be controlled, for example, by changing plasmid copy numbers in synthetic systems.

The reaction flux vector 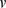 contains all the reactions in the system, that is, binding/unbinding and protein production/decay. Given that binding/unbinding reactions occur on a much faster time-scale than protein production/decay [1], we partition 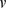 into 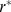 and 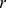, where 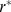 contains the slow processes, whereas 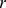 is composed of the fast reactions, that is,
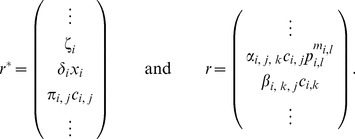
(18)


### Biochemical Parameters

Since the production of a typical protein takes approximately 5 minutes [1], and a few dozen mRNAs can be transcribed from the same gene simultaneously by [49], and similarly, a few dozen proteins can be translated from the same mRNA at the same time by [50], the effective production rate of protein from a gene can be as high as 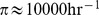. This value can be arbitrarily decreased, for instance, by decreasing the RBS strength in synthetic circuits. The cell volume of *Escherichia coli* is typically between 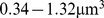 by [51], so that 1 molecule/cell corresponds to approximately 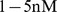 concentration. By [52], a typical value of the dissociation constant of bacterial promoters is 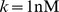, whereas [22] suggests 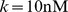, and experimentally obtained values are provided in [53]. One of the most widely used high copy number vectors is the pUC plasmid [54], which can have hundreds of copies per cell [55]. A frequently used medium copy number plasmid is p15A with a few dozen copies per cell [56], whereas pSC101 is regarded as a low copy number plasmid with only a few copies per cell [56]. Finally, since the lifetime of a protein is on the order of a cell-cycle [1], we have 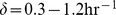 [50]. The typical range of macroscopic parameters in *Escherichia coli* is summarized in Table 1.

**Table 1:** Typical range of macroscopic parameters in *Escherichia coli*.

Parameter	Symbol	Range	Unit	Reference
Production rate constant	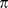	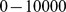	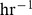	[1], [49], [50]
Dissociation constant	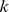	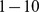	nM	[22], [52], [53]
DNA concentration	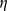	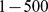	nM	[51], [54], [56], [56]
Protein decay rate	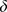	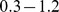	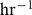	[1], [50]

If we had not neglected mRNA dynamics, there would be three different time scales in the system. Binding and unbinding reactions occur on the time scale of seconds (or even subseconds) [1], representing the fastest time scale. The intermediate time scale is that of mRNA dynamics, as the average lifetime of mRNA is on the time scale of minutes [57], [58], [59]. Finally, the dynamics of proteins evolve on the slowest time scale (hours). As we are interested in describing the dynamics of the system on the time scale of gene expression, the concentration of promoter complexes and mRNA transcripts can be both approximated with their quasi-steady state values, leading to the models we have proposed in the paper. However, we would like to point out that including mRNA dynamics would not change anything substantial in the results and it would simply add 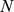 more ODEs to the ODE model of an 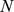-node module without any effects on the retroactivity matrices (shown in [8] considering a specific example).

### Definition of 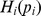 and 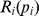


First, note that 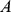 in (2) has a block diagonal structure, yielding
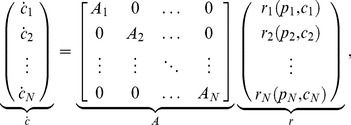
(19)where 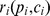 denotes the reaction flux vector corresponding to reversible binding reactions with the promoter of 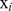. Let 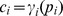 denote the vector of concentrations of complexes with the promoter of 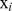 at the quasi-steady state, obtained by setting 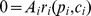.

We first define 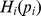 as follows:
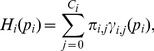
(20)where 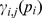 is the 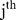 entry in 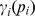 and 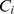 is the number of complexes with the promoter of 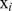.

Next, define the matrix 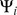 as follows: it has as many columns as the number of complexes formed with the promoter of 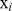, and as many rows as the number of parents of 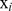:
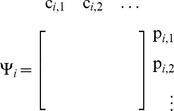
such that its 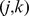 element is 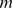 if the 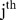 parent of 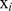 is bound as an 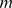-multimer in 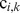 (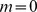 if the 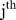 parent is not bound). Finally, for nodes having parents, define the *retroactivity* 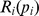 *of node* 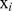 as
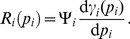
(21)For the most common binding types, 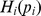 and 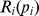 are given in Figure 3. For details on their derivation, see Supporting Text S3.

## Supporting Information

Figure S1
**Mixing retroactivity can be avoided by introducing an extra node.** Rectangles represent promoter regions, arrows denote coding regions. Promoters are regulated by TFs expressed from coding regions of the same color. (A) The production of 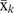 is regulated by two TFs: 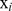 from the module, and 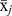 from the context. If the binding of 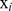 is not completely independent from that of 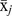, the mixing retroactivity 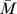 of the context is non-zero. As a result, the dynamics of the context can suppress that of the module by (12). (B) One possible solution to obtain zero mixing retroactivity 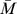 is to introduce an extra input node 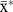 in the context, so that parents from the module and from the context are not mixed. In particular, replace the promoter of 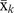 with one that is regulated by 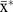. As a result, parents from the module and from the context are not mixed anymore, yielding 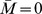, in the meantime, 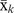 still integrates the signal coming from 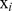 (through 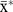) and from 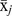.(EPS)

Text S1 **ODE model of the system in** **Figure 2** **together with the parameter values used for simulation.**
(PDF)

Text S2 **Appendix containing the Theorems and Propositions together with the corresponding proofs.** Subsections include the following: (1) Isolated module without input; (2) Isolated module with input; (3) Interconnection of modules; and (4) Bounding the difference between the trajectories of an isolated and a connected module.
(PDF)

Text S3
**Derivation of** 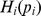 **and** 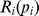 **for the most common binding types presented in** **Figure 3** **.**
(PDF)
