# Supplementary material for: Modular Composition of Gene Transcription Networks
Source: PLoS Comput Biol. 2014 Mar 13;10(3):e1003486. doi: 10.1371/journal.pcbi.1003486 (PMC3952816; doi:10.1371/journal.pcbi.1003486)
Supplement: Text S1 — ODE model of the system in Figure 2 together with the parameter values used for simulation. (PDF) [file pcbi.1003486.s002.pdf]

Text S1

Here, we provide the ODE model of the system in Figure 2 together with the parameter values used for simulation.

Consider first the isolated module in Figure 2A. The reversible binding reactions in the module are given by

$$\mathrm{c}_{1,0} + n\mathrm{x}_1 \underset{\beta_1}{\overset{\alpha_1}{\rightleftharpoons}} \mathrm{c}_{1,1},$$

where $\mathrm{c}_{1,0}$ and $\mathrm{c}_{1,1}$ denote the complexes of the promoter of x_1 without and with x_1 bound as an n-multimer, respectively. Protein production and decay are modeled by

$$\varnothing \underset{\delta_1}{\overset{\zeta_1}{\rightleftharpoons}} \mathrm{x}_1, \qquad \mathrm{c}_{1,0} \xrightarrow{\pi_{1,0}} \mathrm{c}_{1,0} + \mathrm{x}_1, \qquad \mathrm{c}_{1,1} \xrightarrow{\pi_{1,1}} \mathrm{c}_{1,1} + \mathrm{x}_1,$$

together with the conservation law $\eta_1 = c_{1,0} + c_{1,1}$, yielding $c_{1,0} = \eta_1 - c_{1,1}$. As a result, referring to (2), with $x = x_1$ and $c = c_{1,1}$ we have

$$A = \begin{pmatrix} 1 & -1 \end{pmatrix}, \qquad B = \begin{pmatrix} -n & n \end{pmatrix}, \qquad B^* = \begin{pmatrix} 1 & -1 & 1 & 1 \end{pmatrix},$$

$$r(x,c) = \begin{pmatrix} \alpha_1 c_{1,0} x_1^n \\ \beta_1 c_{1,1} \end{pmatrix}, \qquad r^*(x,c) = \begin{pmatrix} \zeta_1 \\ \delta_1 x_1 \\ \pi_{1,0} c_{1,0} \\ \pi_{1,1} c_{1,1} \end{pmatrix},$$

yielding $g(x,c) = \zeta_1 - \delta_1 x_1 + \pi_{1,0} c_{1,0} + \pi_{1,1} c_{1,1}$. Note that r does not depend on u as the module has no input (u is vacuous). When the module is interconnected to its context (Figure 2C), we have the following reversible binding reactions in the case when x_1 and $\bar{\mathrm{x}}_1$ are competing for the same binding sites of the promoter of $\bar{\mathrm{x}}_2$:

$$\bar{\mathrm{c}}_{2,0} + \bar{n}\mathrm{x}_1 \underset{\bar{\beta}_1}{\overset{\bar{\alpha}_1}{\rightleftharpoons}} \bar{\mathrm{c}}_{2,1}, \qquad \bar{\mathrm{c}}_{2,0} + \bar{m}\bar{\mathrm{x}}_1 \underset{\bar{\beta}_2}{\overset{\bar{\alpha}_2}{\rightleftharpoons}} \bar{\mathrm{c}}_{2,2},$$

where $\bar{\mathrm{c}}_{2,0}$, $\bar{\mathrm{c}}_{2,1}$ and $\bar{\mathrm{c}}_{2,2}$ denote the empty promoter of $\bar{\mathrm{x}}_2$, and the promoter complexes with x_1 as an $\bar{n}$-multimer and with $\bar{\mathrm{x}}_1$ as an $\bar{m}$-multimer, respectively. Protein production and decay in the context are modeled by

$$\varnothing \underset{\bar{\delta}_1}{\overset{\bar{\zeta}_1}{\rightleftharpoons}} \bar{\mathrm{x}}_1, \qquad \bar{\mathrm{c}}_{1,0} \xrightarrow{\bar{\pi}_{1,0}} \bar{\mathrm{c}}_{1,0} + \bar{\mathrm{x}}_1, \qquad \bar{\mathrm{c}}_{2,0} \xrightarrow{\bar{\pi}_{2,0}} \bar{\mathrm{c}}_{2,0} + \bar{\mathrm{x}}_2,$$

$$\varnothing \underset{\bar{\delta}_2}{\overset{\bar{\zeta}_2}{\rightleftharpoons}} \bar{\mathrm{x}}_2, \qquad \bar{\mathrm{c}}_{2,1} \xrightarrow{\bar{\pi}_{2,1}} \bar{\mathrm{c}}_{2,1} + \bar{\mathrm{x}}_2, \qquad \bar{\mathrm{c}}_{2,2} \xrightarrow{\bar{\pi}_{2,2}} \bar{\mathrm{c}}_{2,2} + \bar{\mathrm{x}}_2,$$

together with the conservation laws $\bar{\eta}_1 = \bar{c}_{1,0}$ and $\bar{\eta}_2 = \bar{c}_{2,0} + \bar{c}_{2,1} + \bar{c}_{2,2}$, yielding $\bar{c}_{2,0} = \bar{\eta}_2 - \bar{c}_{2,1} - \bar{c}_{2,2}$. Referring to (4), with $\bar{x} = \begin{pmatrix} \bar{x}_1 & \bar{x}_2 \end{pmatrix}^T$, $\bar{c} = \begin{pmatrix} \bar{c}_{2,1} & \bar{c}_{2,2} \end{pmatrix}^T$ and $\bar{u} = x_1$ we have

$$\bar{E} = \begin{pmatrix} -\bar{n} & \bar{n} & 0 & 0 \end{pmatrix}, \qquad \bar{r}(\bar{x}, \bar{c}, \bar{u}) = \begin{pmatrix} \bar{\alpha}_1 \bar{c}_{2,0} x_1^{\bar{n}} \\ \bar{\beta}_1 \bar{c}_{2,1} \\ \bar{\alpha}_2 \bar{c}_{2,0} \bar{x}_1^{\bar{m}} \\ \bar{\beta}_2 \bar{c}_{2,2} \end{pmatrix},$$

yielding $s(\bar{x}, \bar{c}, \bar{u}) = \bar{E}\bar{r}(\bar{x}, \bar{c}, \bar{u})$. The dynamics of the context ($\bar{c}$ and $\bar{x}$) are given by (3) with

$$\bar{A} = \begin{bmatrix} 1 & -1 & 0 & 0 \\ 0 & 0 & 1 & -1 \end{bmatrix}, \qquad \bar{B}^* = \begin{bmatrix} 1 & -1 & 1 & 0 & 0 & 0 & 0 & 0 \\ 0 & 0 & 0 & 1 & -1 & 1 & 1 & 1 \end{bmatrix},$$

$$\bar{r}^{*} = \begin{pmatrix} \bar{\zeta}_1 & \bar{\delta}_1\bar{x}_1 & \bar{\pi}_{1,0}c_{1,0} & \bar{\zeta}_2 & \bar{\delta}_2\bar{x}_2 & \bar{\pi}_{2,0}\bar{c}_{2,0} & \bar{\pi}_{2,1}\bar{c}_{2,1} & \bar{\pi}_{2,2}\bar{c}_{2,2} \end{pmatrix}^T,$$

$$E = \begin{pmatrix} 0 \\ 0 \end{pmatrix}, \qquad \bar{B} = \begin{bmatrix} 0 & 0 & -\bar{n} & \bar{n} \\ 0 & 0 & 0 & 0 \end{bmatrix}.$$

Simulation parameters in Figure 2 are as follows: $\eta_1 = \bar{\eta}_1 = \bar{\eta}_2 = 50\text{nM}$, $\delta_1 = \bar{\delta}_1 = \bar{\delta}_2 = 1\text{hr}^{-1}$, $\zeta_1(t) = 100 + 100\cos(2\pi t)\,[\text{nM hr}^{-1}]$, $\bar{\zeta}_1(t) = 100 + 100\cos(\pi t)\,[\text{nM hr}^{-1}]$, $\pi_{1,0} = \pi_{1,1} = \bar{\pi}_{1,0} = \bar{\pi}_{2,0} = \bar{\pi}_{2,1} = \bar{\pi}_{2,2} = 0$, $n = \bar{n} = \bar{m} = 4$, $\alpha_1 = \bar{\alpha}_1 = \bar{\alpha}_2 = 100\text{nM}^{-4}\ \text{hr}^{-1}$ and $\beta_1 = \bar{\beta}_1 = \bar{\beta}_2 = 100\text{hr}^{-1}$.
