# Supplementary material for: Modular Composition of Gene Transcription Networks
Source: PLoS Comput Biol. 2014 Mar 13;10(3):e1003486. doi: 10.1371/journal.pcbi.1003486 (PMC3952816; doi:10.1371/journal.pcbi.1003486)
Supplement: Text S2 — Appendix containing the Theorems and Propositions together with the corresponding proofs. Subsections include the following: (1) Isolated module without input; (2) Isolated module with input; (3) Interconnection of modules; and (4) Bounding the difference between the trajectories of an isolated and a connected module. (PDF) [file pcbi.1003486.s003.pdf]

Text S2

Here, we provide the Theorems and Propositions together with the corresponding proofs. Subsections include the following: (1) Isolated module without input; (2) Isolated module with input; (3) Interconnection of modules; and (4) Bounding the difference between the trajectories of an isolated and a connected module.

Isolated module without input

In this section, we consider a module with no input. Introduce

$$x_B(c) := \sum_{\{\, i \,|\, \mathrm{x}_i \in \Phi \,\}} V_i^T \Psi_i c_i, \tag{S1}$$

the concentration vector of bound internal TFs in the module. That is, the ith entry of x_B is the concentration of x_i bound in promoter complexes. Furthermore, define $x_T := x + x_B$, the vector of total concentrations of internal TFs (free and bound).

Proposition 1. *With $x_T = x + x_B$ we obtain $\dot{x}_T = g\,(x, c)$.*

Proof. Considering the block diagonal structure of A in (19) yields

$$\dot{x}_T = g(x, c) + Br + \sum_{\{\, i \,|\, \mathrm{x}_i \in \Phi \,\}} V_i^T \Psi_i A_i r_i$$

from (2) and (S1). Therefore, it is enough to prove that

$$Br = - \sum_{\{\, i \,|\, \mathrm{x}_i \in \Phi \,\}} V_i^T \Psi_i A_i r_i = - \underbrace{\left[\; V_1^T \Psi_1 A_1 \;\middle|\; V_2^T \Psi_2 A_2 \;\middle|\; \dots \;\right]}_{\widetilde{B}} r. \tag{S2}$$

As a result, it is sufficient to show that $B = -\widetilde{B}$. We prove this by focusing on the first block $V_1^T \Psi_1 A_1$ of $\widetilde{B}$ in (S2), since the procedure can be repeated for the other blocks the same way.

Recall that

$$V_1^T = \begin{array}{c} \begin{array}{ccc} \mathrm{p}_{1,1} & \mathrm{p}_{1,2} & \cdots \end{array} \\ \left[\begin{array}{ccc} \; & \; & \; \\ \; & \; & \; \\ \; & \; & \; \end{array} \right] \end{array} \begin{array}{l} \mathrm{x}_1 \\ \mathrm{x}_2 \\ \vdots \end{array} \quad \text{and} \quad \Psi_1 = \begin{array}{c} \begin{array}{ccc} \mathrm{c}_{1,1} & \mathrm{c}_{1,2} & \cdots \end{array} \\ \left[\begin{array}{ccc} \; & \; & \; \\ \; & \; & \; \\ \; & \; & \; \end{array} \right] \end{array} \begin{array}{l} \mathrm{p}_{1,1} \\ \mathrm{p}_{1,2} \\ \vdots \end{array} ,$$

where the entries are defined as follows. In the binary matrix V_1^T an entry is 1 if the species labeling the corresponding row and column are the same, otherwise the entry is 0. An entry in Ψ_1 is m if the promoter complex labeling the corresponding column has m molecules bound of the parent labeling the corresponding row. As a result, an entry in

$$V_1^T \Psi_1 = \begin{array}{c} \begin{array}{ccc} \mathrm{c}_{1,1} & \mathrm{c}_{1,2} & \cdots \end{array} \\ \left[\begin{array}{ccc} \; & \; & \; \\ \; & \; & \; \\ \; & \; & \; \end{array} \right] \end{array} \begin{array}{l} \mathrm{x}_1 \\ \mathrm{x}_2 \\ \vdots \end{array} ,$$

is m if the promoter complex labeling the corresponding column has m molecules bound of the TF labeling the corresponding row (we used the fact that V_1 is binary and there is at most a single 1 in each row and column). Furthermore, note that

$$A_1 = \begin{array}{c} \rho_1 \quad \rho_2 \quad \cdots \\ \left[\begin{array}{ccc} & & \\ & & \\ & & \end{array} \right] \end{array} \begin{array}{l} \mathrm{c}_{1,1} \\ \mathrm{c}_{1,2} \\ \vdots \end{array} ,$$

such that an entry in A_1 is 1 (or -1) if the reversible binding reaction ρ_l labeling the column releases (or sequesters) the promoter complex labeling the corresponding row. If the reaction does not affect the concentration of the promoter complex, the corresponding entry is 0. As a result, the (j,k) entry in $V_1^T \Psi_1 A_1$ being n means the following. Upon occurrence of the reversible binding reaction ρ_k, the number of x_j bound to promoter complexes changes with n, that is, the concentration of free x_j changes with $-n$. Similarly to A_1, we have

$$B = \begin{array}{c} \rho_1 \quad \rho_2 \quad \cdots \\ \left[\begin{array}{ccc} & & \\ & & \\ & & \end{array} \right] \end{array} \begin{array}{l} \mathrm{x}_1 \\ \mathrm{x}_2 \\ \vdots \end{array} ,$$

such that the (j,k) entry in B being equal to n^* means the following. If $n^* > 0$, the reaction releases n^* molecules of x_j; if $n^* < 0$, the reaction sequesters n^* molecules of x_j; and if $n^* = 0$, the reaction does not affect the concentration of x_j. That is, the concentration of free x_j changes with n^* upon occurrence of the reversible binding reaction ρ_k. As a result, we conclude that the (j,k) entry of B is equal to the negative of the (j,k) entry in $V_1^T \Psi_1 A_1$, which completes the proof. □

Proposition 2. *Let $c = \gamma(x)$ be the locally unique solution of $0 = Ar(x,c)$. When $c = \gamma(x)$, we obtain $\dot{x}_T = h(x)$ with $h(x)$ given in (6).*

Proof. Proposition 1 yields $\dot{x}_T = g(x,c)$, therefore, it is sufficient to show that $h(x) = g(x, \gamma(x))$. According to Detailed Description of the System Model in Methods, we can write $g(x,c) = B^* r^*$ in (2) as

$$g(x,c) = \begin{pmatrix} \zeta_1 + \sum_{j=1}^{C_1} \pi_{1,j} c_{1,j} - \delta_1 x_1 \\ \zeta_2 + \sum_{j=1}^{C_2} \pi_{2,j} c_{2,j} - \delta_2 x_2 \\ \vdots \\ \zeta_N + \sum_{j=1}^{C_N} \pi_{N,j} c_{N,j} - \delta_N x_N \end{pmatrix}, \tag{S3}$$

where C_i is the number of complexes with the promoter of x_i. Next, consider the block diagonal structure of A in (19), and let $c_i = \gamma_i(p_i)$ be the locally unique solution of $0 = A_i r_i(p_i, c_i)$. Then, we can write the manifold $c = \gamma(x)$ as $c_i = \gamma_i(p_i)$ for $i = 1, 2, \ldots, N$. With this, we obtain that when $c = \gamma(x)$, we have

$$g(x,\gamma(x)) = \begin{pmatrix} \zeta_1 + \sum_{j=1}^{C_1} \pi_{1,j} \gamma_{1,j}(p_1) - \delta_1 x_1 \\ \zeta_2 + \sum_{j=1}^{C_2} \pi_{2,j} \gamma_{2,j}(p_2) - \delta_2 x_2 \\ \vdots \\ \zeta_N + \sum_{j=1}^{C_N} \pi_{N,j} \gamma_{N,j}(p_N) - \delta_N x_N \end{pmatrix},$$

where $\gamma_{i,j}(p_i)$ is the jth entry in $\gamma_i(p_i)$. With (20), this yields $g(x,\gamma(x)) = h(x)$, which completes the proof. □

Proposition 3. *Define* $R(x) := \frac{\mathrm{d}x_B(\gamma(x))}{\mathrm{d}x}$ *and consider* $R_i(p_i)$ *defined in* (21)*. Then, we have*

$$R(x) = \sum_{\{\,i\,|\,\mathrm{x}_i \in \Phi\,\}} V_i^T R_i(p_i) V_i.$$

Proof. Consider the block diagonal structure of A in (19), and let $c_i = \gamma_i(p_i)$ be the locally unique solution of $0 = A_i r_i(p_i, c_i)$. Then, we can write the manifold $c = \gamma(x)$ as $c_i = \gamma_i(p_i)$ for $i = 1, 2, \ldots, N$. Given that $p_i = V_i x$ if the module has no input, we have $\frac{\mathrm{d}p_i}{\mathrm{d}x} = V_i$. Then, using the chain rule and (S1), we obtain

$$R(x) = \sum_{\{\,i\,|\,\mathrm{x}_i \in \Phi\,\}} V_i^T \Psi_i \frac{\mathrm{d}\gamma_i(p_i)}{\mathrm{d}x} = \sum_{\{\,i\,|\,\mathrm{x}_i \in \Phi\,\}} V_i^T \Psi_i \frac{\mathrm{d}\gamma_i(p_i)}{\mathrm{d}p_i}\frac{\mathrm{d}p_i}{\mathrm{d}x} = \sum_{\{\,i\,|\,\mathrm{x}_i \in \Phi\,\}} V_i^T R_i(p_i) V_i.$$

□

According to [29], the binding and unbinding reactions are much faster than protein production and decay. By picking some nonzero decay rate δ and dissociation rate constant β, the dimensionless parameter $\epsilon := \delta/\beta \ll 1$ captures the difference between the two time scales. Note that r in (18) can be written as

$$r = \begin{pmatrix} \vdots \\ \alpha_{i,j,k} c_{i,j} p_{i,l}^{m_{i,l}} \\ \beta_{i,k,j} c_{i,k} \\ \vdots \end{pmatrix} = \frac{\beta}{\delta}\begin{pmatrix} \vdots \\ \delta \frac{\alpha_{i,j,k}}{\beta} c_{i,j} p_{i,l}^{m_{i,l}} \\ \delta \frac{\beta_{i,k,j}}{\beta} c_{i,k} \\ \vdots \end{pmatrix} = \frac{1}{\epsilon}\underbrace{\begin{pmatrix} \vdots \\ \delta \frac{\alpha_{i,j,k}}{\beta} c_{i,j} p_{i,l}^{m_{i,l}} \\ \delta \frac{\beta_{i,k,j}}{\beta} c_{i,k} \\ \vdots \end{pmatrix}}_{\widehat{r}} = \frac{1}{\epsilon}\widehat{r}. \tag{S4}$$

Let $(c(t), x(t))$ be the solution of the isolated module dynamics (2) for $t \in [0, t_f]$ with initial condition (c_0, x_0). Let $c = \widetilde{\gamma}(x_T)$ be an isolated root of $0 = Ar(x_T - x_B(c), c)$, and define $w := c - \widetilde{\gamma}(x_T)$, $\tau := t/\epsilon$ and the dynamics

$$\dot{x} = [I + R(x)]^{-1} h(x). \tag{S5}$$

Let $\widehat{x}(t)$ be the solution of (S5) for $t \in [0, t_f]$ with initial condition $\widehat{x}(0) = \widehat{x}_0$ such that $\widehat{x}_0 + x_B(\gamma(\widehat{x}_0)) = x_0 + x_B(c_0)$.

Theorem 1. *Consider* $\frac{\mathrm{d}w}{\mathrm{d}\tau} = A\widehat{r}(x_T - x_B(w + \widetilde{\gamma}(x_T)), w + \widetilde{\gamma}(x_T))$ *and assume that* $w = 0$ *is an exponentially stable equilibrium point uniformly in* x_T *and let* $\mathcal{C}$ *be a compact subset of the region of attraction. If* $c_0 \in \mathcal{C}$*, then there exists a constant* $\epsilon^* > 0$ *such that for* $0 < \epsilon < \epsilon^*$ *we have* $\|x(t) - \widehat{x}(t)\|_2 = O(\epsilon)$ *for* $t \in [0, t_f]$*.*

Proof. Since we can write $\dot{x}_T = g(x, c)$ with $x_T = x + x_B(c)$ according to Proposition 1, we can transform (2) into standard singular perturbation form [1] using (S4) with $x_T(0) := x_0 + x_B(\gamma(x_0))$:

$$\dot{x}_T = g(x_T - x_B(c), c), \tag{S6}$$

$$\epsilon\dot{c} = A\widehat{r}(x_T - x_B(c), c). \tag{S7}$$

To obtain the reduced order model, we set $\epsilon = 0$ in (S7). Since $0 = A\widehat{r}(x_T - x_B(c), c)$ implies $0 = Ar(x_T - x_B(c), c) = Ar(x, c)$, we conclude that the slow manifold is given by $c = \widetilde{\gamma}(x_T) = \gamma(x)$. Therefore, the reduced system becomes $\dot{x}_T = g(x, \gamma(x)) = h(x)$ by Proposition 2. As $x_T = x + x_B(c)$, on the slow manifold we have $\dot{x}_T = \dot{x} + \frac{\mathrm{d}x_B(\gamma(x))}{\mathrm{d}x}\dot{x} = [I + R(x)]\dot{x}$, equivalent to (S5). Applying Theorem 11.1 in [1] concludes the proof. □

Isolated module with input

Here, we consider a module with inputs, so that we now have $r(x, c, u)$ instead of $r(x, c)$, unlike in the previous section. As before, internal TFs of the module do not participate in binding/unbinding reactions outside the module.

Proposition 4. *Let $c = \gamma(x, u)$ be the locally unique solution of $0 = Ar(x, c, u)$ and define $R(x, u) := \frac{\partial x_B(\gamma(x,u))}{\partial x}$ and $Q(x, u) := \frac{\partial x_B(\gamma(x,u))}{\partial u}$. Then, we have*

$$R(x, u) = \sum_{\{\, i \,|\, \mathrm{x}_i \in \Phi \,\}} V_i^T R_i(p_i) V_i \qquad \textit{and} \qquad Q(x, u) = \sum_{\{\, i \,|\, \mathrm{x}_i \in \Phi \cap \Omega \,\}} V_i^T R_i(p_i) D_i.$$

Proof. Consider the block diagonal structure of A in (19), and let $c_i = \gamma_i(p_i)$ be the locally unique solution of $0 = A_i r_i(p_i, c_i)$. Then, we can write the manifold $c = \gamma(x)$ as $c_i = \gamma_i(p_i)$ for $i = 1, 2, \ldots, N$. Given that

$$p_i = \left[\begin{array}{cc} V_i & D_i \end{array}\right] \left(\begin{array}{c} x \\ u \end{array}\right),$$

we have $\frac{\partial p_i}{\partial x} = V_i$ and $\frac{\partial p_i}{\partial u} = D_i$. Then, using the chain rule and (S1), we obtain

$$R(x, u) = \sum_{\{\, i \,|\, \mathrm{x}_i \in \Phi \,\}} V_i^T \Psi_i \frac{\partial \gamma_i(p_i)}{\partial x} = \sum_{\{\, i \,|\, \mathrm{x}_i \in \Phi \,\}} V_i^T \Psi_i \frac{\mathrm{d}\gamma_i(p_i)}{\mathrm{d}p_i} \frac{\partial p_i}{\partial x} = \sum_{\{\, i \,|\, \mathrm{x}_i \in \Phi \,\}} V_i^T R_i(p_i) V_i.$$

Similarly, we obtain

$$Q(x, u) = \sum_{\{\, i \,|\, \mathrm{x}_i \in \Phi \,\}} V_i^T \Psi_i \frac{\partial \gamma_i(p_i)}{\partial u} = \sum_{\{\, i \,|\, \mathrm{x}_i \in \Phi \,\}} V_i^T \Psi_i \frac{\mathrm{d}\gamma_i(p_i)}{\mathrm{d}p_i} \frac{\partial p_i}{\partial u} = \sum_{\{\, i \,|\, \mathrm{x}_i \in \Phi \cap \Omega \,\}} V_i^T R_i(p_i) D_i,$$

where we used the fact that $D_i = 0$ if $\mathrm{x}_i \notin \Omega$. □

Remark. Note that $R(x, u)$ in Proposition 4 can be computed the same way as $R(x)$ in Proposition 3, the only difference is their argument. This is due to the fact that when the module has input u, some of the parents of internal TFs are external TFs, which means that the retroactivity of the node x_i depends on p_i, which is a function of both x and u. △

Similarly to before, let $(c(t), x(t))$ be the solution of the isolated module dynamics (2) for $t \in [0, t_f]$ with initial condition (c_0, x_0) and with smooth input $u(t)$. Note that $\hat{r}$ defined in (S4) is now a function of x, c and u, however, for simpler notation, we write it as $\hat{r}(x, c, t)$. Let $c = \tilde{\gamma}(x_T, u)$ be an isolated root of $0 = Ar(x_T - x_B(c), c, u)$, and define $\hat{\gamma}(x_T, t) := \tilde{\gamma}(x_T, u)$, $w := c - \hat{\gamma}(x_T, t)$, $h(x, u) := g(x, \gamma(x, u))$, $\tau := t/\epsilon$, together with the dynamics

$$\dot{x} = [I + R(x, u)]^{-1} [h(x, u) - Q(x, u)\dot{u}] =: f(x, u, \dot{u}). \tag{S8}$$

Let $\hat{x}(t)$ be the solution of (S8) for $t \in [0, t_f]$ with initial condition $\hat{x}(0) = \hat{x}_0$ such that $\hat{x}_0 + x_B(\gamma(\hat{x}_0, u(0))) = x_0 + x_B(c_0)$.

Theorem 2. *Consider $\frac{\mathrm{d}w}{\mathrm{d}\tau} = A\hat{r}(x_T - x_B(w + \hat{\gamma}(x_T, t)), w + \hat{\gamma}(x_T, t), t)$ and assume that $w = 0$ is an exponentially stable equilibrium point uniformly in (x_T, t) and let $\mathcal{C}$ be a compact subset of the region of attraction. If $c_0 \in \mathcal{C}$, then there exists a constant $\epsilon^* > 0$ such that for $0 < \epsilon < \epsilon^*$ we have $\|x(t) - \hat{x}(t)\|_2 = O(\epsilon)$ for $t \in [0, t_f]$.*

Proof. Since we can write $\dot{x}_T = g(x,c)$ with $x_T = x + x_B(c)$ according to Proposition 1, we can transform (2) into standard singular perturbation form using (S4) with $x_T(0) := x_0 + x_B(\gamma(x_0, u(0)))$:

$$\dot{x}_T = g\left(x_T - x_B(c), c\right), \tag{S9}$$
$$\epsilon\dot{c} = A\hat{r}\left(x_T - x_B(c), c, t\right). \tag{S10}$$

To obtain the reduced order model, we set $\epsilon = 0$ in (S10). Since $0 = A\hat{r}\left(x_T - x_B(c), c, t\right)$ implies $0 = Ar\left(x_T - x_B(c), c, u\right) = Ar(x,c,u)$, we conclude that the slow manifold [1] is given by $c = \tilde{\gamma}(x_T, u) = \gamma(x,u)$. Therefore, the reduced system becomes $\dot{x}_T = g(x, \gamma(x,u)) = h(x,u)$ from Proposition 2 (since r is a function of u, $h(.)$ is also a function of u). As $x_T = x + x_B$, on the slow manifold we have $\dot{x}_T = \dot{x} + \frac{\partial x_B(\gamma(x,u))}{\partial x}\dot{x} + \frac{\partial x_B(\gamma(x,u))}{\partial u}\dot{u} = \left[I + R(x,u)\right]\dot{x} + Q(x,u)\dot{u}$, equivalent to (S8). Applying Theorem 11.1 in [1] concludes the proof. □

Interconnection of modules

In this section we consider the interconnection of the module Σ and its context. Recall that $u = U\bar{x}$ is the input to Σ, and define $q(c) := \sum_{\{\, i \,|\, \mathrm{x}_i \in \Phi \,\}} [D_i U]^T \Psi_i c_i$, so that q_j denotes the concentration of bound u_j (TF from the context) in Σ.

Proposition 5. *Let $c = \gamma(x,u)$ be the locally unique solution of $0 = Ar(x,c,u)$ and define $S(x,\bar{x}) := \frac{\partial q(\gamma(x,U\bar{x}))}{\partial \bar{x}}$ and $M(x,\bar{x}) := \frac{\partial q(\gamma(x,U\bar{x}))}{\partial x}$. Then, we can compute S and M as*

$$S(x,\bar{x}) = \sum_{\{\, i \,|\, \mathrm{x}_i \in \Omega \,\}} \left[D_i U\right]^T R_i(p_i) D_i U \qquad \text{and} \qquad M(x,\bar{x}) = \sum_{\{\, i \,|\, \mathrm{x}_i \in (\Phi \cap \Omega) \,\}} \left[D_i U\right]^T R_i(p_i) V_i.$$

Proof. Consider the block diagonal structure of A in (19), and let $c_i = \gamma_i(p_i)$ be the locally unique solution of $0 = A_i r_i(p_i, c_i)$. Then, we can write the manifold $c = \gamma(x)$ as $c_i = \gamma_i(p_i)$ for $i = 1, 2, \ldots, N$. Given that

$$p_i = \begin{bmatrix} V_i & D_i \end{bmatrix} \begin{pmatrix} x \\ u \end{pmatrix},$$

we have $\frac{\partial p_i}{\partial x} = V_i$ and $\frac{\partial p_i}{\partial u} = D_i$, and finally, $u = U\bar{x}$ yields $\frac{\mathrm{d}u}{\mathrm{d}\bar{x}} = U$. Then, using the chain rule and (S1), we obtain

$$S(x,\bar{x}) = \sum_{\{\, i \,|\, \mathrm{x}_i \in \Omega \,\}} \left[D_i U\right]^T \Psi_i \frac{\mathrm{d}\gamma_i(p_i)}{\mathrm{d}p_i} \frac{\partial p_i}{\partial u} \frac{\mathrm{d}u}{\mathrm{d}\bar{x}} = \sum_{\{\, i \,|\, \mathrm{x}_i \in \Omega \,\}} \left[D_i U\right]^T R_i(p_i) D_i U,$$

and similarly, we have

$$M(x,\bar{x}) = \sum_{\{\, i \,|\, \mathrm{x}_i \in \Omega \,\}} \left[D_i U\right]^T \Psi_i \frac{\mathrm{d}\gamma_i(p_i)}{\mathrm{d}p_i} \frac{\partial p_i}{\partial x} = \sum_{\{\, i \,|\, \mathrm{x}_i \in \Omega \,\}} \left[D_i U\right]^T R_i(p_i) V_i = \sum_{\{\, i \,|\, \mathrm{x}_i \in (\Phi \cap \Omega) \,\}} \left[D_i U\right]^T R_i(p_i) V_i,$$

where we used the fact that $V_i = 0$ for $\mathrm{x}_i \notin \Phi$. □

The isolated dynamics of Σ and that of its context are given by

$$\begin{aligned} \dot{c} &= Ar(x,c,u), & \dot{x} &= g(x,c) + Br(x,c,u), \\ \dot{\bar{c}} &= \bar{A}\bar{r}(\bar{x},\bar{c},\bar{u}), & \dot{\bar{x}} &= \bar{g}(\bar{x},\bar{c}) + \bar{B}\bar{r}(\bar{x},\bar{c},\bar{u}) \end{aligned} \tag{S11}$$

by (2), respectively. Once we insert Σ into its context, however, their dynamics change according to (4), yielding

$$\begin{aligned} \dot{c} &= Ar(x,c,U\bar{x}), & \dot{x} &= g(x,c) + Br(x,c,U\bar{x}) + s\left(\bar{x},\bar{c},\bar{U}x\right), \\ \dot{\bar{c}} &= \bar{A}\bar{r}\left(\bar{x},\bar{c},\bar{U}x\right), & \dot{\bar{x}} &= \bar{g}(\bar{x},\bar{c}) + \bar{B}\bar{r}\left(\bar{x},\bar{c},\bar{U}x\right) + \bar{s}(x,c,U\bar{x}), \end{aligned} \tag{S12}$$

with $s = \bar{E}\bar{r}\left(\bar{x}, \bar{c}, \bar{U}x\right)$ and $\bar{s} = Er\left(x, c, U\bar{x}\right)$. When considering the interconnection of Σ with its context, it is useful to treat them as one module in isolation, which we call Σ'. Therefore, we define

$$\begin{aligned} x' &:= \left(\begin{array}{c} x \\ \bar{x} \end{array} \right), \qquad c' := \left(\begin{array}{c} c \\ \bar{c} \end{array} \right), \qquad g'(x', c') := \left(\begin{array}{c} g(x, c) \\ \bar{g}(\bar{x}, \bar{c}) \end{array} \right), \\ r'(x', c') &:= \left(\begin{array}{c} r(x, c, U\bar{x}) \\ \bar{r}(\bar{x}, \bar{c}, \bar{U}x) \end{array} \right), \qquad A' := \begin{bmatrix} A & 0 \\ 0 & \bar{A} \end{bmatrix}, \qquad B' := \begin{bmatrix} B & \bar{E} \\ E & \bar{B} \end{bmatrix}. \end{aligned} \tag{S13}$$

Consequently, (S12) can be rewritten as the isolated module dynamics (2) with (S13). Finally introduce

$$G := \begin{bmatrix} (I + R)^{-1} & 0 \\ 0 & (I + \bar{R})^{-1} \end{bmatrix}, \qquad \Delta := \begin{bmatrix} \bar{S} & \overline{M} \\ M & S \end{bmatrix}, \qquad \Xi := \begin{bmatrix} 0 & QU \\ \bar{Q}\bar{U} & 0 \end{bmatrix}, \tag{S14}$$

where R, Q, S and M are the internal, external, scaling and mixing retroactivity matrices of Σ, respectively, and similarly for the context.

Proposition 6. *The internal retroactivity R' of Σ' can be written as $R = G^{-1} - I + \Delta + \Xi$.*

Proof. Recall that according to Proposition 3, the internal retroactivity $R'(x')$ of Σ' can be calculated as $R' = \sum_{\left\{ i \,|\, \mathrm{x}_i' \in \Phi' \right\}} [V_i']^T R_i'(p_i') V_i'$, where R_i' is the retroactivity of node x_i' and Φ' is the set of nodes having parents in Σ'. Note that $\mathrm{x}_i' = \mathrm{x}_i$ for $i = 1, 2, \dots, N$ and $\mathrm{x}_{N+j}' = \bar{\mathrm{x}}_j$ for $j = 1, 2, \dots, \bar{N}$. That is, according to (S13), in Σ' first come the nodes in the module Σ followed by the nodes in the context.

We first show that $V_i' = \left[\begin{array}{cc} V_i & D_i U \end{array} \right]$ for nodes that are in Σ ($i = 1, 2, \dots, N$). To this end, recall that the binary matrix V_i' establishes the relationship between the parents of node x_i' and the nodes in Σ':

$$V_i' = \begin{array}{c} \mathrm{x}_1' \;\; \mathrm{x}_2' \;\; \dots \;\; \mathrm{x}_N' \;\;\; \mathrm{x}_{N+1}' \;\; \mathrm{x}_{N+2}' \;\; \dots \;\; \mathrm{x}_{N+\overline{N}}' \\ \left[\begin{array}{c|c} \qquad\qquad & \qquad\qquad \\ & \\ & \end{array} \right] \end{array} \begin{array}{l} \\ \mathrm{p}_{i,1}' \\ \mathrm{p}_{i,2}' \\ \vdots \end{array}$$

where an entry is 1 if the species indexing the corresponding row and column are the same, otherwise the entry is zero. Exploiting the order of the nodes, we can relabel the rows and columns of V_i' as

$$V_i' = \begin{array}{c} \mathrm{x}_1 \;\; \mathrm{x}_2 \;\; \dots \;\; \mathrm{x}_N \;\;\; \bar{\mathrm{x}}_1 \;\; \bar{\mathrm{x}}_2 \;\; \dots \;\; \bar{\mathrm{x}}_{\overline{N}} \\ \left[\begin{array}{c|c} \qquad\qquad & \qquad\qquad \\ & \\ & \end{array} \right] \end{array} \begin{array}{l} \\ \mathrm{p}_{i,1} \\ \mathrm{p}_{i,2} \\ \vdots \end{array} . \tag{S15}$$

Given that the rows and columns of V_i are labeled as

$$V_i = \begin{array}{c} \mathrm{x}_1 \;\; \mathrm{x}_2 \;\; \dots \;\; \mathrm{x}_N \\ \left[\begin{array}{c} \qquad\qquad \\ \\ \end{array} \right] \end{array} \begin{array}{l} \\ \mathrm{p}_{i,1} \\ \mathrm{p}_{i,2} \\ \vdots \end{array} ,$$

we conclude that the first block in V_i' is equivalent to V_i. Next, we show that the second block in V_i' is equivalent to $D_i U$. To this end, recall that

$$D_i = \begin{array}{c} \mathrm{u}_1 \;\; \mathrm{u}_2 \;\; \dots \\ \left[\begin{array}{c} \qquad\qquad \\ \\ \end{array} \right] \end{array} \begin{array}{l} \\ \mathrm{p}_{i,1} \\ \mathrm{p}_{i,2} \\ \vdots \end{array} \quad \text{and} \quad U = \begin{array}{c} \bar{\mathrm{x}}_1 \;\; \bar{\mathrm{x}}_2 \;\; \dots \;\; \bar{\mathrm{x}}_{\overline{N}} \\ \left[\begin{array}{c} \qquad\qquad \\ \\ \end{array} \right] \end{array} \begin{array}{l} \\ \mathrm{u}_1 \\ \mathrm{u}_2 \\ \vdots \end{array} ,$$

yielding

$$D_iU = \begin{array}{c} \begin{matrix} \bar{\mathrm{x}}_1 & \bar{\mathrm{x}}_2 & \dots & \bar{\mathrm{x}}_{\bar{N}} \end{matrix} \\ \left[\begin{matrix} \quad & \quad & \quad & \quad \\ \quad & \quad & \quad & \quad \\ \quad & \quad & \quad & \quad \end{matrix} \right] \end{array} \begin{matrix} \mathrm{p}_{i,1} \\ \mathrm{p}_{i,2} \\ \vdots \end{matrix} \;,$$

exploiting the fact that D_i and U are binary, furthermore, that there is at most a single 1 in each row and column. As a result, we conclude that the second block of V_i' in (S15) is D_iU, so that we obtain $V_i' = \left[\begin{array}{cc} V_i & D_iU \end{array}\right]$ for nodes that are in Σ $(i = 1, 2, \dots, N)$. Similarly, we obtain $V_{N+j}' = \left[\begin{array}{cc} \bar{D}_j\bar{U} & \bar{V}_j \end{array}\right]$ for nodes in the context $(j = 1, 2, \dots, \bar{N})$.

Next, note that the set of nodes in Σ having parents is given by $\Phi \cup \Omega$, as the parents are either from Σ or they are inputs of Σ. Similarly, the set of nodes in the context having parents is given by $\bar{\Phi} \cup \bar{\Omega}$. Consequently, the set of nodes in Σ' having parents is given by $\Phi' = \Phi \cup \Omega \cup \bar{\Phi} \cup \bar{\Omega}$. Furthermore, we have $R_i'\left(p_i'\right) = R_i\left(p_i\right)$ for $i = 1, 2, \dots, N$ and $R_{N+j}'\left(p_j'\right) = \bar{R}_j\left(\bar{p}_j\right)$ for $j = 1, 2, \dots, \bar{N}$. As a result, the internal retroactivity R' of Σ' can be written as

$$\begin{aligned} R' &= \sum_{\left\{ i \,|\, \mathrm{x}_i \in (\Phi \cup \Omega) \right\}} [V_i']^T R_i' V_i' + \sum_{\left\{ j \,|\, \bar{\mathrm{x}}_j \in \left(\bar{\Phi} \cup \bar{\Omega}\right) \right\}} [V_{N+j}']^T R_{N+j}' V_{N+j}' \\ &= \sum_{\left\{ i \,|\, \mathrm{x}_i \in (\Phi \cup \Omega) \right\}} [V_i']^T R_i V_i' + \sum_{\left\{ j \,|\, \bar{\mathrm{x}}_j \in \left(\bar{\Phi} \cup \bar{\Omega}\right) \right\}} [V_{N+j}']^T \bar{R}_j V_{N+j}'. \end{aligned} \tag{S16}$$

Employing $V_i' = \left[\begin{array}{cc} V_i & D_iU \end{array}\right]$ for $i = 1, 2, \dots, N$ yields

$$\sum_{\left\{ i \,|\, \mathrm{x}_i \in (\Phi \cup \Omega) \right\}} [V_i']^T R_i V_i' = \sum_{\left\{ i \,|\, \mathrm{x}_i \in (\Phi \cup \Omega) \right\}} \left[\begin{array}{cc} V_i^T R_i V_i & V_i^T R_i D_i U \\ \left[D_iU\right]^T R_i V_i & \left[D_iU\right]' R_i D_i U \end{array}\right]. \tag{S17}$$

Next, we focus on each of the four block matrices on the right hand side of (S17). Recall that $V_i = 0$ if $\mathrm{x}_i \notin \Phi$, and similarly, $D_i = 0$ if $\mathrm{x}_i \notin \Omega$. We can write the upper left block of (S17) as

$$\sum_{\{ i \,|\, \mathrm{x}_i \in (\Phi \cup \Omega) \}} V_i^T R_i V_i = \sum_{\{ i \,|\, \mathrm{x}_i \in \Phi \}} V_i^T R_i V_i + \sum_{\{ i \,|\, \mathrm{x}_i \in (\Omega \setminus \Phi) \}} V_i^T R_i V_i = \sum_{\{ i \,|\, \mathrm{x}_i \in \Phi \}} V_i^T R_i V_i = R \tag{S18}$$

by Proposition 3. Similarly, the upper right block matrix on the right hand side of (S17) can be written as

$$\begin{aligned} \sum_{\{ i \,|\, \mathrm{x}_i \in (\Phi \cup \Omega) \}} V_i^T R_i D_i U = & \sum_{\{ i \,|\, \mathrm{x}_i \in (\Phi \setminus \Omega) \}} V_i^T R_i D_i U + \sum_{\{ i \,|\, \mathrm{x}_i \in (\Omega \setminus \Phi) \}} V_i^T R_i D_i U \\ & + \sum_{\{ i \,|\, \mathrm{x}_i \in (\Phi \cap \Omega) \}} V_i^T R_i D_i U = \sum_{\{ i \,|\, \mathrm{x}_i \in (\Phi \cap \Omega) \}} V_i^T R_i D_i U = QU \end{aligned} \tag{S19}$$

by Proposition 4. The lower left block matrix on the right hand side of (S17) reduces to

$$\begin{aligned} \sum_{\{ i \,|\, \mathrm{x}_i \in (\Phi \cup \Omega) \}} \left[D_iU\right]^T R_i V_i = & \sum_{\{ i \,|\, \mathrm{x}_i \in (\Phi \setminus \Omega) \}} \left[D_iU\right]^T R_i V_i + \sum_{\{ i \,|\, \mathrm{x}_i \in (\Omega \setminus \Phi) \}} \left[D_iU\right]^T R_i V_i \\ & + \sum_{\{ i \,|\, \mathrm{x}_i \in (\Phi \cap \Omega) \}} \left[D_iU\right]^T R_i V_i = \sum_{\{ i \,|\, \mathrm{x}_i \in (\Phi \cap \Omega) \}} \left[D_iU\right]^T R_i V_i = M \end{aligned} \tag{S20}$$

according to Proposition 5, and similarly, the lower right block matrix in (S17) reads

$$\begin{aligned} \sum_{\{ i \,|\, \mathrm{x}_i \in (\Phi \cup \Omega) \}} \left[D_iU\right]^T R_i D_i U = & \sum_{\{ i \,|\, \mathrm{x}_i \in \Omega \}} \left[D_iU\right]^T R_i D_i U + \sum_{\{ i \,|\, \mathrm{x}_i \in (\Phi \setminus \Omega) \}} \left[D_iU\right]^T R_i D_i U \\ = & \sum_{\{ i \,|\, \mathrm{x}_i \in \Omega \}} \left[D_iU\right]^T R_i D_i U = S. \end{aligned} \tag{S21}$$

Therefore, combining (S17) with (S18)–(S21), we obtain

$$\sum_{\{\,i\,|\,\mathrm{x}_i\in\Phi\cup\Omega\,\}} \left[V_i'\right]^T R_i V_i' = \left[\begin{array}{cc} R & QU \\ M & S \end{array}\right], \tag{S22}$$

and similarly, one can show that

$$\sum_{\left\{\,j\,|\,\bar{\mathrm{x}}_j\in\bar{\Phi}\cup\bar{\Omega}\,\right\}} \left[V_{N+j}'\right]^T \bar{R}_j V_{N+j}' = \left[\begin{array}{cc} \bar{S} & \overline{M} \\ \bar{Q}\bar{U} & \bar{R} \end{array}\right]. \tag{S23}$$

Substituting (S22)–(S23) into (S16) yields

$$R' = \left[\begin{array}{cc} R & QU \\ M & S \end{array}\right] + \left[\begin{array}{cc} \bar{S} & \overline{M} \\ \bar{Q}\bar{U} & \bar{R} \end{array}\right],$$

so that

$$R' = \left[\begin{array}{cc} I+R & 0 \\ 0 & I+\bar{R} \end{array}\right] - \left[\begin{array}{cc} I & 0 \\ 0 & I \end{array}\right] + \left[\begin{array}{cc} \bar{S} & \overline{M} \\ M & S \end{array}\right] + \left[\begin{array}{cc} 0 & QU \\ \bar{Q}\bar{U} & 0 \end{array}\right] = G^{-1} - I + \Delta + \Xi.$$

□

Proposition 7. *Let* $c' = \gamma'(x')$ *be the locally unique solution of* $0 = L'r'(x',c')$ *and define* $h'(x') := g'(x', \gamma'(x'))$*. Then, the right-hand side of*

$$\dot{x}' = (I + R')^{-1} h'(x') \tag{S24}$$

is equivalent to the right-hand side of (12)*. As a result, a solution of* (12) *satisfies* (S24) *with identical initial conditions, and vice versa.*

Proof. Let $c = \gamma(x,u)$ and $\bar{c} = \bar{\gamma}(\bar{x},\bar{u})$ denote the locally unique solution of $0 = Ar(x,c,u)$ and $0 = \bar{A}\bar{r}(\bar{x},\bar{c},\bar{u})$, respectively. Since $u = U\bar{x}$ and $\bar{u} = \bar{U}x$ upon interconnection, the block diagonal structure of A' in (S13) yields

$$\gamma'(x') = \left(\begin{array}{c} \gamma(x, U\bar{x}) \\ \bar{\gamma}(\bar{x}, \bar{U}x) \end{array}\right),$$

and similarly, considering $h(x,u) = g(x,\gamma(x,u))$ and $\bar{h}(\bar{x},\bar{u}) = \bar{g}(\bar{x},\bar{\gamma}(\bar{x},\bar{u}))$ together with (S13) results in

$$h'(x') = \left(\begin{array}{c} h(x, U\bar{x}) \\ \bar{h}(\bar{x}, \bar{U}x) \end{array}\right).$$

According to Theorem 2, the isolated dynamics of Σ and that of its context are well approximated by

$$\begin{aligned} \dot{x} &= [I+R]^{-1}\left[h(x,u) - Q\dot{u}\right] =: f(x,u,\dot{u}), \\ \dot{\bar{x}} &= \left[I+\bar{R}\right]^{-1}\left[\bar{h}(\bar{x},\bar{u}) - \bar{Q}\dot{\bar{u}}\right] =: \bar{f}(\bar{x},\bar{u},\dot{\bar{u}}). \end{aligned} \tag{S25}$$

Rewriting this with G and Ξ defined in (S14) yields

$$h'(x') = G^{-1}\left(\begin{array}{c} f(x, U\bar{x}) \\ \bar{f}(\bar{x}, \bar{U}x) \end{array}\right) + \Xi\dot{x}'.$$

Substituting this into $\dot{x}' = (I+R')^{-1}h'(x')$ and using $R = G^{-1} - I + \Delta + \Xi$ from Proposition 6 yields

$$\dot{x}' = \left(G^{-1} + \Delta + \Xi\right)^{-1} G^{-1}\left(\begin{array}{c} f\left(x, U\bar{x}, U\dot{\bar{x}}\right) \\ \bar{f}\left(\bar{x}, \bar{U}x, \bar{U}\dot{x}\right) \end{array}\right) + \left(G^{-1} + \Delta + \Xi\right)^{-1}\Xi\dot{x}'$$

which reduces to

$$\dot{x}' = \left[I - \left(G^{-1} + \Delta + \Xi\right)^{-1}\Xi\right]^{-1}\left(G^{-1} + \Delta + \Xi\right)^{-1}G^{-1}\begin{pmatrix} f\left(x, U\bar{x}, U\dot{\bar{x}}\right) \\ \bar{f}\left(\bar{x}, \bar{U}x, \bar{U}\dot{x}\right). \end{pmatrix}. \tag{S26}$$

Given that

$$\left(G^{-1} + \Delta + \Xi\right)^{-1}G^{-1} = \left[G\left(G^{-1} + \Delta + \Xi\right)\right]^{-1} = \left(I + G\Delta + G\Xi\right)^{-1},$$

we can write (S26) as

$$\begin{aligned}
\dot{x}' =& \left[I - \left(I + G\Delta + G\Xi\right)^{-1}G\Xi\right]^{-1}\left(I + G\Delta + G\Xi\right)^{-1}\begin{pmatrix} f\left(x, U\bar{x}, U\dot{\bar{x}}\right) \\ \bar{f}\left(\bar{x}, \bar{U}x, \bar{U}\dot{x}\right) \end{pmatrix} \\
=& \left\{\left(I + G\Delta + G\Xi\right)\left[I - \left(I + G\Delta + G\Xi\right)^{-1}G\Xi\right]\right\}^{-1}\begin{pmatrix} f\left(x, U\bar{x}, U\dot{\bar{x}}\right) \\ \bar{f}\left(\bar{x}, \bar{U}x, \bar{U}\dot{x}\right) \end{pmatrix} \\
=& \left(I + G\Delta + G\Xi - G\Xi\right)^{-1}\begin{pmatrix} f\left(x, U\bar{x}, U\dot{\bar{x}}\right) \\ \bar{f}\left(\bar{x}, \bar{U}x, \bar{U}\dot{x}\right) \end{pmatrix} \\
=& \left(I + G\Delta\right)^{-1}\begin{pmatrix} f\left(x, U\bar{x}, U\dot{\bar{x}}\right) \\ \bar{f}\left(\bar{x}, \bar{U}x, \bar{U}\dot{x}\right) \end{pmatrix},
\end{aligned}$$

which is equivalent to (12). □

Let $(c'(t), x'(t))$ be the solution of the isolated module dynamics (2) with (S13) for $t \in [0, t_f]$ with initial condition (c'_0, x'_0). Furthermore, let x'_B and x'_T denote the vectors of concentrations of bound and total TFs in Σ', respectively. Let $c' = \widetilde{\gamma}'(x'_T)$ be an isolated root of $0 = A'r'(x'_T - x'_B(c'), c')$, and define $w' := c' - \widetilde{\gamma}'(x'_T)$, $\tau := t/\epsilon$. Let $\widehat{x}'(t)$ be the solution of (12) for $t \in [0, t_f]$ with initial condition $\widehat{x}'(0) = \bar{x}'_0$ such that $\widehat{x}'_0 + x'_B(\gamma'(\widehat{x}'_0)) = x'_0 + x'_B(c'_0)$.

Theorem 3. *Consider $\frac{\mathrm{d}w'}{\mathrm{d}\tau} = A\widehat{r}'\left(x'_T - x'_B(w' + \widetilde{\gamma}'(x'_T)), w' + \widetilde{\gamma}'(x'_T)\right)$ and assume that $w' = 0$ is an exponentially stable equilibrium point uniformly in x'_T and let $\mathcal{C}$ be a compact subset of the region of attraction. If $c'_0 \in \mathcal{C}$, then there exists a constant $\epsilon^* > 0$ such that for $0 < \epsilon < \epsilon^*$ we have $\|x'(t) - \widehat{x}'(t)\|_2 = O(\epsilon)$ for $t \in [0, t_f]$.*

Proof. According to Proposition 7, $\widehat{x}'(t)$ is also a solution of $\dot{x}' = [I + R'(x')]^{-1}h'(x')$. Then, by applying Theorem 1 to Σ', that is, to (2) with (S13), we conclude the proof. □

Bounding the difference between the trajectories of an isolated and a connected module

Let

$$\dot{x} = f(x, u, \dot{u}) \tag{S27}$$

denote the dynamics of the module in isolation by (10). Once the module is connected to its context, its dynamics change according to

$$\dot{x} = \left[I + (I + R)^{-1}\,\overline{S}\right]^{-1} f\left(x, u, \dot{u}\right) \tag{S28}$$

by (14), provided that $\overline{M} = 0$. Let $x(t)$ and $\widetilde{x}(t)$ denote the solution of (S27) and (S28), respectively, with identical initial conditions.

Proposition 8. *Consider $\mu(x, u)$ defined in (16). Assume that (i) $f(x, u, \dot{u})$ have Lipschitz constant $\widehat{l}$, (ii) $\|f(x, u, \dot{u})\|_2 \leqslant \widehat{f}$ and (iii) $\mu(x, u) \leqslant \widehat{\mu}$ for $x \in \mathcal{D}_1$, $u \in \mathcal{D}_2$ and $\dot{u} \in \mathcal{D}_3$. If $x(t), \widetilde{x}(t) \in \mathcal{D}_1$, $u \in \mathcal{D}_2$ and $\dot{u} \in \mathcal{D}_3$ for $t \in [0, T]$, then we have $\|x(t) - \widetilde{x}(t)\|_2 \leqslant \frac{\widehat{\mu}\widehat{f}}{\widehat{l}}\left[e^{\widehat{l}t} - 1\right]$ for $t \in [0, T]$.*

Proof. We can rewrite (S28) as

$$\dot{x} = f(x) + \left[\left(I + (I+R)^{-1}\bar{S}\right)^{-1} - I\right] f(x, u, \dot{u}).$$

Furthermore, if $x \in \mathcal{D}_1$, $u \in \mathcal{D}_2$ and $\dot{u} \in \mathcal{D}_3$, the sub-multiplicative property of the induced 2-norm yields

$$\left\| \left[\left(I + (I+R)^{-1}\bar{S}\right)^{-1} - I\right] f(x, u, \dot{u}) \right\|_2 \leqslant \hat{\mu}\hat{f}.$$

Given that the initial conditions of (S27) and (S28) are identical, applying Theorem 3.4 in [1] concludes the proof. □

Moreover, we can provide a constant bound independent of time if the isolated dynamics of the module has an additional property called contraction [2]. A system $\dot{x} = f(x,t)$ is called contracting if there exists a square matrix $\Theta(x,t)$ with the following two properties: (i) $\Theta^T\Theta$ is uniformly positive definite and (ii) the symmetric part of the generalized Jacobian

$$J(x,t) := \left(\dot{\Theta} + \Theta\frac{\partial f}{\partial x}\right)\Theta^{-1}$$

is uniformly negative definite. The absolute value of the largest eigenvalue of the symmetric part of J is called the system's contraction rate with respect to the metric Θ.

Proposition 9. *Assume that (i) the system* (S27) *is contracting with rate $\lambda > 0$ and metric transformation $\Theta(x,t)$, (ii) $\|f(x,u,\dot{u})\|_2 \leqslant \hat{f}$ and (iii) $\mu(x,u) \leqslant \hat{\mu}$ for $x \in \mathcal{D}_1$, $u \in \mathcal{D}_2$ and $\dot{u} \in \mathcal{D}_3$. Denote by $\kappa(x,t)$ the condition number of $\Theta(x,t)$, and let $\hat{\kappa} \geqslant 0$ such that $\hat{\kappa} \geqslant \kappa(x,t)$ for $x \in \mathcal{D}_1$ and for $t \in [0,\infty]$. If $\tilde{x}(t) \in \mathcal{D}_1$, $u \in \mathcal{D}_2$ and $\dot{u} \in \mathcal{D}_3$ for $t \in [0,\infty]$, then we have $\|x(t) - \tilde{x}(t)\|_2 \leqslant \frac{\hat{\mu}\hat{f}\hat{\kappa}}{\lambda}$ for $t \in [0,\infty)$.*

Proof. Rewrite (S28) as

$$\dot{x} = f(x, u, \dot{u}) + \left[\left(I + (I+R)^{-1}\bar{S}\right)^{-1} - I\right] f(x, u, \dot{u}). \quad \text{(S29)}$$

Using the sub-multiplicative property of the induced 2-norm, we have

$$\left\| \left[\left(I + (I+R)^{-1}\bar{S}\right)^{-1} - I\right] f(x, u, \dot{u}) \right\|_2 \leqslant \hat{\mu}\hat{f}$$

for $x(t) \in \mathcal{D}_1$, $u \in \mathcal{D}_2$ and $\dot{u} \in \mathcal{D}_3$. Since $\tilde{x}(t) \in \mathcal{D}_1$ for all t, we apply Lemma 1 in [3] with (S27) as the nominal system and (S29) as the perturbed system. □

Proposition 10. *Let $\sigma_{\min}(I+R)$ denote the smallest singular value of $(I+R)$, and similarly, $\sigma_{\max}(\bar{S})$ stands for the greatest singular value of $\bar{S}$. Then, we have $\mu \leqslant \frac{\sigma_{\max}(\bar{S})}{\sigma_{\min}(I+R) - \sigma_{\max}(\bar{S})}$ for μ defined in* (16) *if $\sigma_{\max}(\bar{S}) < \sigma_{\min}(I+R)$.*

Proof. Let $G := (I+R)^{-1}$, so that $\|G\|_2 = \sigma_{\max}[(I+R)^{-1}] = 1/\sigma_{\min}(I+R)$. Consider the eigenvalue λ of $G\bar{S}$ with the corresponding eigenvector v:

$$|\lambda|\,\|v\|_2 = \|\lambda v\|_2 = \left\|G\bar{S}v\right\|_2 \leqslant \|G\|_2 \left\|\bar{S}\right\|_2 \|v\|_2 = \frac{\sigma_{\max}(\bar{S})}{\sigma_{\min}(I+R)}\|v\|_2 < \|v\|_2,$$

so that $|\lambda| < 1$, consequently, the spectral radius ρ of $G\bar{S}$ satisfies $\rho < 1$. Using the result on the convergence of geometric series of matrices in [4], we can write

$$\left(I + G\bar{S}\right)^{-1} = I + \sum_{k=1}^{\infty}(-1)^k \left(G\bar{S}\right)^k,$$

so that we have

$$\mu \leqslant \sum_{k=1}^{\infty} \left\|G\bar{S}\right\|_2^k = \frac{\left\|G\bar{S}\right\|_2}{1-\left\|G\bar{S}\right\|_2} \leqslant \frac{\|G\|_2\|\bar{S}\|_2}{1-\|G\|_2\|\bar{S}\|_2} = \frac{\sigma_{\max}(\bar{S})}{\sigma_{\min}(I+R)-\sigma_{\max}(\bar{S})},$$

where we used the fact that $\left\|G\bar{S}\right\|_2 \leqslant \|G\|_2 \left\|\bar{S}\right\|_2 = \frac{\sigma_{\max}(\bar{S})}{\sigma_{\min}(I+R)} < 1$. □

References

[1] Khalil HK (2002) Nonlinear systems. Prentice Hall.

[2] Lohmiller W, Slotine JJE (1998) On contraction analysis for non-linear systems. Automatica 34: 683–696.

[3] Del Vecchio D, Slotine JJE (2013) A contraction theory approach to singularly perturbed systems. IEEE Transactions on Automatic Control 58: 752–757.

[4] Strang G (2003) Introduction to linear algebra. SIAM.
