# Supplementary material for: Modular Composition of Gene Transcription Networks
Source: PLoS Comput Biol. 2014 Mar 13;10(3):e1003486. doi: 10.1371/journal.pcbi.1003486 (PMC3952816; doi:10.1371/journal.pcbi.1003486)
Supplement: Text S3 — Derivation of and for the most common binding types presented in Figure 3 . (PDF) [file pcbi.1003486.s004.pdf]

Text S3

Here, we provide the details of the derivation of $H_i(p_i)$ and $R_i(p_i)$ for the most common binding types presented in Figure 3.

We first consider the case when x_i has no parents, so that there are no reversible binding reactions with the promoter of x_i, yielding $\eta_i = c_{i,0}$, and thus $H_i = \pi_{i,0}\eta_i$ by (20). Furthermore, R_i is not defined for nodes without parents.

Next, we focus on the case when x_i has a single parent y, that is, $p_i = y$. The binding of y to the empty promoter $\mathrm{c}_{i,0}$ of x_i as an n-multimer forms the promoter complex $\mathrm{c}_{i,1}$, given by the reaction

$$\mathrm{c}_{i,0} + n\mathrm{y} \underset{\beta_y}{\overset{\alpha_y}{\rightleftharpoons}} \mathrm{c}_{i,1},$$

together with the conservation law $\eta_i = c_{i,0} + c_{i,1}$, resulting in $c_{i,0} = \eta_1 - c_{i,1}$. As a result, we have

$$\dot{c}_i = \underbrace{\begin{pmatrix} 1 & -1 \end{pmatrix}}_{A_i} \underbrace{\begin{pmatrix} \alpha_y c_{i,0} y^n \\ \beta_y c_{i,1}, \end{pmatrix}}_{r_i},$$

so that $c_i = \gamma_i(y)$ denotes the solution of the above equation when the left-hand side is set to zero, corresponding to the value of $c_{i,1}$ at the quasi-steady state. Substituting this into (20) yields the corresponding $H_i(y)$ in Figure 3. Similarly, substituting $\gamma_i(y)$ into (21) together with $\Psi_i = n$ results in the corresponding $R_i(y)$ in Figure 3.

Next, take node x_i, coregulated by two TFs, y and z, so that $p_i = \begin{pmatrix} y & z \end{pmatrix}^T$. Furthermore, for y and z, denote the corresponding multimerization factors by n and m, the association constant rates by α_y and α_z, and the dissociation constant rates by β_y and β_z, respectively. Let $k_y = \beta_y/\alpha_y$ and $k_z = \beta_z/\alpha_z$ denote the dissociation constant of y and z to the promoter of x_i, respectively.

In the case of independent binding, the parents bind to different sites of the promoter, not affecting each other's binding/unbinding, represented by the reactions

$$\mathrm{c}_{i,0} + n\mathrm{y} \underset{\beta_y}{\overset{\alpha_y}{\rightleftharpoons}} \mathrm{c}_{i,1}, \qquad \mathrm{c}_{i,0} + m\mathrm{z} \underset{\beta_z}{\overset{\alpha_z}{\rightleftharpoons}} \mathrm{c}_{i,2}, \qquad \mathrm{c}_{i,2} + n\mathrm{y} \underset{\beta_y}{\overset{\alpha_y}{\rightleftharpoons}} \mathrm{c}_{i,3}, \qquad \mathrm{c}_{i,1} + m\mathrm{z} \underset{\beta_z}{\overset{\alpha_z}{\rightleftharpoons}} \mathrm{c}_{i,3},$$

together with the conservation law $\eta_i = c_{i,0} + c_{i,1} + c_{i,2} + c_{1,3}$, yielding $c_{i,0} = \eta_i - c_{i,1} - c_{i,2} - c_{1,3}$, so that

$$\underbrace{\begin{pmatrix} \dot{c}_{i,1} \\ \dot{c}_{i,2} \\ \dot{c}_{i,3} \end{pmatrix}}_{\dot{c}_i} = \underbrace{\begin{bmatrix} 1 & -1 & 0 & 0 & -1 & 1 & 0 & 0 \\ 0 & 0 & 1 & -1 & 0 & 0 & -1 & 1 \\ 0 & 0 & 0 & 0 & 1 & -1 & 1 & -1 \end{bmatrix}}_{A_i} \underbrace{\begin{pmatrix} \alpha_y c_{i,0} y^n \\ \beta_y c_{i,1} \\ \alpha_z c_{i,0} z^m \\ \beta_z c_{i,2} \\ \alpha_z c_{i,1} z^m \\ \beta_z c_{i,3} \\ \alpha_y c_{i,2} y^n \\ \beta_y c_{i,3} \end{pmatrix}}_{r_i}$$

where $c_i = \gamma_i(y, z)$ denotes the solution of the above set of equations when the left-hand side is set to zero. Substituting this into (20) yields the corresponding $H_i(y, z)$ in Figure 3. Similarly, substituting $\gamma_i(y, z)$ into (21) together with

$$\Psi_i = \begin{bmatrix} n & 0 & n \\ 0 & m & m \end{bmatrix}.$$

results in the corresponding $R_i(y, z)$ in Figure 3.

In the case of competitive binding, the parents are competing for the same binding sites which they occupy mutually exclusively. As a result, complex $\mathrm{c}_{i,3}$ is not formed, represented by the reactions

$$\mathrm{c}_{i,0} + n\mathrm{y} \underset{\beta_y}{\overset{\alpha_y}{\rightleftharpoons}} \mathrm{c}_{i,1}, \qquad \mathrm{c}_{i,0} + m\mathrm{z} \underset{\beta_z}{\overset{\alpha_z}{\rightleftharpoons}} \mathrm{c}_{i,2},$$

together with the conservation law $\eta_i = c_{i,0} + c_{i,1} + c_{i,2}$, yielding $c_{i,0} = \eta_i - c_{i,1} - c_{i,2}$, so that

$$\underbrace{\begin{pmatrix} \dot{c}_{i,1} \\ \dot{c}_{i,2} \end{pmatrix}}_{\dot{c}_i} = \underbrace{\begin{bmatrix} 1 & -1 & 0 & 0 \\ 0 & 0 & 1 & -1 \end{bmatrix}}_{A_i} \underbrace{\begin{pmatrix} \alpha_y c_{i,0} y^n \\ \beta_y c_{i,1} \\ \alpha_z c_{i,0} z^m \\ \beta_z c_{i,2} \end{pmatrix}}_{r_i}$$

where $c_i = \gamma_i(y,z)$ denotes the solution of the above set of equations when the left-hand side is set to zero. Substituting this into (20) yields the corresponding $H_i(y,z)$ in Figure 3. Similarly, substituting $\gamma_i(y,z)$ into (21) together with

$$\Psi_i = \begin{bmatrix} n & 0 \\ 0 & m \end{bmatrix}$$

results in the corresponding $R_i(y,z)$ in Figure 3.

In the case of cooperative binding, z can only bind after y, that is, complex $\mathrm{c}_{i,2}$ is not formed, represented by the reactions

$$\mathrm{c}_{i,0} + n\mathrm{y} \underset{\beta_y}{\overset{\alpha_y}{\rightleftharpoons}} \mathrm{c}_{i,1}, \qquad \mathrm{c}_{i,1} + m\mathrm{z} \underset{\beta_z}{\overset{\alpha_z}{\rightleftharpoons}} \mathrm{c}_{i,3},$$

together with the conservation law $\eta_i = c_{i,0} + c_{i,1} + c_{1,3}$, yielding $c_{i,0} = \eta_i - c_{i,1} - c_{1,3}$, so that

$$\underbrace{\begin{pmatrix} \dot{c}_{i,1} \\ \dot{c}_{i,3} \end{pmatrix}}_{\dot{c}_i} = \underbrace{\begin{bmatrix} 1 & -1 & -1 & 1 \\ 0 & 0 & 1 & -1 \end{bmatrix}}_{A_i} \underbrace{\begin{pmatrix} \alpha_y c_{i,0} y^n \\ \beta_y c_{i,1} \\ \alpha_z c_{i,1} z^m \\ \beta_z c_{i,3} \end{pmatrix}}_{r_i}$$

where $c_i = \gamma_i(y,z)$ denotes the solution of the above set of equations when the left-hand side is set to zero. Substituting this into (20) yields the corresponding $H_i(y,z)$ in Figure 3. Similarly, substituting $\gamma_i(y,z)$ into (21) together with

$$\Psi_i = \begin{bmatrix} n & n \\ 0 & m \end{bmatrix}$$

results in the corresponding $R_i(y,z)$ in Figure 3.
